# Improving Autism Diagnosis Across Ages Using Eye-Tracking and Temporal Transformer Models

**DOI:** 10.3390/s26165302

**Published:** 2026-08-21

**Authors:** Mohammed A. AlZain, Mahmoud Rokaya, Dalia I. Hemdan, Ibrahim Gad, Malik Almaliki, Elsayed Atlam

**Affiliations:** 1 Department of Information Technology, College of Computers and Information Technology, Taif University, Taif 21944, Saudi Arabia; 2 King Salman Center for Disability Research, Riyadh 11614, Saudi Arabia; dalia.m@tu.edu.sa; 3 Department Computer Science Faculty of Science, Tanta University, Tanta 31527, Egypt; 4 Department of Food Science and Nutrition, Faculty of Science, Taif University, Taif 21944, Saudi Arabia; 5 Department of Computer Science, College of Computer Science and Engineering, Taibah University, Yanbu 46421, Saudi Arabia

**Keywords:** autism spectrum disorder (ASD), eye-tracking, gaze biomarkers, temporal transformer, entropy-based modeling, attention mechanisms, cross-age generalization, cross-dataset transfer, probabilistic sequence modeling, explainable artificial intelligence (XAI), neurodevelopmental diagnostics

## Abstract

Variation in gaze behavior due to age is currently a considerable challenge in building reliable eye-tracking systems for Autism Spectrum Disorder (ASD) diagnosis. However, existing strategies often focus on static gaze representation or dataset-based information, which can lead to limited generalization of findings depending on developmental groups and heterogeneous recording conditions. In this paper, we present a temporal transformer-based system for ASD classification using eye-tracking sequences. This allows you to model gaze behavior as a structured temporal process in the context of contextual attention, as well as employing entropy-based modeling for various distributions of variability over time and temporal consistency constraints to capture sequential gaze dynamics related to ASD behavioral patterns. The framework was evaluated using public eye-tracking corpus containing temporally ordered gaze recordings from ASD and TD participants across age groups. Five sequential experiments on baseline classification, class-balancing analysis, cross-age evaluation, ablation analysis, and cross-dataset transfer learning were performed to conduct experiment-based evaluations. Model performed 0.91 in in-domain Area Under the Receiver Operating Characteristic Curve (AUC) and 0.81 in F1-score on the primary eye-tracking dataset. In the cross-dataset assessment stage, the framework presented a relatively stable performance, with an AUC of 0.85 and an average F1-score of 0.74, irrespective of differences in participant distributions and recording conditions. Ablation analysis also revealed that entropy regularization and temporal consistency mechanisms played a significant role in model stability and classification performance. The ablation analysis provides additional insight into the contribution of the proposed framework components beyond the overall classification performance. Removing the entropy-based regularization reduced the model’s ability to represent variability in gaze allocation, whereas removing the temporal-consistency regularization resulted in less stable sequence representations during learning. These observations indicate that the proposed components complement the transformer-based sequence encoder by improving representation stability and preserving diagnostically relevant temporal information. Rather than acting as independent classifiers, the regularization mechanisms serve as supporting constraints that enhance the quality and robustness of the learned temporal representations. The results indicate that temporally structured gaze modeling is more robust, interpretable, and general in comparison to static gaze representations. In summary, the presented framework can represent a scalable and developmentally appropriate approach to gaze-based ASD classification and support the implementation of trusted neurodevelopmental screening systems.

## 1. Introduction

Autism Spectrum Disorder (ASD) is a complex neurodevelopmental condition that is characterized by persistent differences in social communication, interaction and patterns of attention. Eye-tracking is one of the promising tools to assess ASD across different developmental stages because visual attention provides a clear view of underlying cognitive and social processes. The variations in gaze behavior, including atypical social attention and visual exploration, have become a primary focus of computational research. Previous studies have found that changes in social orientation, fixation behavior, and visual preference can be used for early ASD screening using eye-tracking data [1,2,3]. However, most existing approaches rely on hand-selected gaze descriptors and traditional machine learning pipelines. While such methods can achieve good statistical performance, they frequently overlook the underlying mechanisms and their deviations under ASD gaze dynamics. The most fundamental limitation of past work lies in viewing gaze data as a static feature set rather than as a structured temporal process. In most cases, gaze sequences are reduced to aggregated descriptors, obscuring the sequential dependencies and temporal variability of the gaze domain. Consequently, these models are non-interpretable and do not provide a principled explanation for the diagnostic information encoding gaze behavior. More fundamentally, the lack of formal mathematical structure constrains the possibility of reasoning about model behavior, generalization characteristics, and robustness across developmental stages.

Eye movements give us easily measurable information on the distribution of visual attention across space and time. The measurements most frequently examined are fixations (which represent periods during which gaze remains relatively stable), saccades (which refer to rapid movements between fixation locations), scan paths (representing the ordered sequence of fixations and transitions), fixation duration, pupil measurements, and AOI (Area of Interest) proportion. These factors offer complementary data on where attention is focused, how long attention is fixed on a location, and how it shifts over time. Eye-tracking studies have often described abnormal focus on socially relevant areas such as eyes, mouths, faces, and human actions in Autism Spectrum Disorder. Differences have also been noted in fixation duration, the structure of scan paths, transitions between social and non-social areas, and the stability of attention across time. These patterns are relevant since they add an objective, non-invasive approach to visual behavior measurement, and can supplement standardized clinical assessment. Information concerning eye movement can also be captured in aggregated indicators (e.g., total fixation duration or proportion of gaze directed toward an AOI) or in ordered temporal sequences. Despite their contribution as information summaries, aggregated indicators might mask the nature of the variation in the order, timing, and recurrence of gaze events. Thus, temporal analysis provides an opportunity to understand not only where participants are looking, but how their gaze changes over time. This distinction is important in ASD, where all participants may have a similar overall fixation distribution, but different temporal patterns of attention allocation.

Recent attempts have employed deep neural networks or ensemble-based methods to enhance classification with gaze-derived features [4,5,6]. Despite generating more complicated patterns, these models are also somewhat unintuitive, especially regarding the temporal logic and the decision space. Most of these models move from input features to predictions with a direct mapping but do not explicitly model the recursive and dynamic nature of gaze behavior. This limitation is all the more pronounced in ASD, where attention is non-linear, temporally variable, and context-specific. To tackle these problems, we offer a unified framework, which combines a mathematically based view of gaze dynamics with a transformer-based implementation model. The formulation presented here takes gaze to be a stochastic temporal process including entropy, attention alignment, and transition dynamics. These components come from proven principles in joint attention and entropy-based modeling [7,8] and provide a mechanism to model variability and uncertainty in the allocation of gazes. To be applied more fully, the mathematical formulation is made consistent with the implementation model which enables a direct correspondence between the theoretical constructs and the computational mechanisms.

Based on this formulation, the implementation model uses a temporal transformer framework to model long-range dependencies in gaze sequences. Attention mechanisms are used to adaptively focus on diagnostically relevant temporal segments, retaining a level of flexibility in modeling non-linear interactions across time. Moreover, this framework includes regularization strategies that maintain stability across age groups and maintain consistency in repeated attention patterns, which further enables the model to generalize more appropriately across developmental settings.

The main contributions of this work can be summarized as follows:

- Proposing a transformer-driven architecture for the modeling of gaze sequences that retains temporal structure, while also serving context-aware representation learning.
- A probabilistic structure based on entropy and attention alignment that allows for a principled interpretation of gaze dynamics beyond traditional feature-based strategies is suggested.
- Demonstrating the clinical value with which the framework for ASD diagnosis is applicable by measuring subtle, developmentally dependent behavioral trends.
- Considering cross-age and cross-dataset generalization to find that the learned representations can be maintained in the presence of differences in demographic distribution and under the circumstances of data acquisition. Although prior work has explored gaze-based classification and deep learning models, little focus has been placed on the incorporation of formal temporal modeling with cross-age generalization in a coherent model.

The rest of this paper is organized as follows. Section 2 reviews related work on eye-tracking and machine learning approaches for ASD diagnosis. Section 3 presents the proposed mathematical formulation, transformer architecture, implementation framework, and evaluation methodology. Section 4 presents the experimental results and a quantitative evaluation across baseline, demographic, ablation, and cross-dataset transfer settings. Section 5 discusses the implications, interpretability, robustness, and limitations of the proposed framework. Finally, Section 6 concludes the paper and outlines future research directions.

## 2. Literature Review

Recent developments in eye-tracking technology as well as machine learning have significantly expanded the potential for autism spectrum disorder (ASD) diagnosis via gaze behavior. An increasing body of evidence also has provided evidence that atypical gaze patterns, including decreased social attention, altered fixation behavior, and preference for non-social stimuli, may represent early indicators of ASD in individuals at multiple developmental stages. Together, these studies demonstrate that gaze-based behavioral phenotyping is a non-invasive, scalable diagnostic tool.

Eye-tracking studies have found several complementary gaze measures that describe different aspects of visual attention. Commonly studied measures are fixations, saccades, scan paths, fixation time, pupil activity, and distribution of gaze in AOIs. Together these measures describe where visual attention is being focused, how long it is maintained and how gaze moves from one target to another. Numerous studies have shown that abnormalities in these measures offer objective behavioral indicators of ASD and complement conventional clinical assessment by identifying atypical patterns of social attention and visual exploration [3,9,10].

Early studies were specifically directed towards behavior marker identification by controlled experimental paradigms. For example, [3] showed that infants with ASD have greater preference of geometric over social stimuli, where gaze preference was strongly associated with symptom severity [1]. Subsequent studies have considered multimodal approaches combining eye-tracking with EEG signals and observed enhanced classification performance in supplementary data sources [11]. Meta-analyses by [9,11] supported the diagnostic significance of gaze features in various experimental contexts [3,12].

As machine learning technology has emerged, a few models have been proposed to automate the detection of ASD from gaze data. Older methods had handcrafted features and classifiers, like Support Vector Machines (SVM), whereas newer works have implemented deep learning architectures, such as Convolutional Neural Networks (CNN), Recurrent Neural Networks (RNN), Long Short-Term Memory (LSTM), and Gated Recurrent Units (GRU). Hybrid models combining CNN and LSTM architectures have demonstrated improved performance by capturing both spatial and temporal characteristics of gaze patterns [4]. Transfer learning approaches and ensemble methods have also been explored to enhance classification accuracy and robustness [6,13]. Related studies have further examined eye-tracking in early autism research and diagnosis [14,15], sensing technologies and algorithmic approaches for early ASD detection [16,17], scan-path biomarkers [18], and the broader contribution of machine learning and eye-tracking technology to ASD research [19].

Concurrently, multiple studies are also working on enhancing the interpretability and clinical importance of gaze-based models. To capture the dynamics of attention and the correlation to clinical assessments such as ADOS-2, recent studies have proposed novel gaze metrics, particularly Favorable AOI Shifts and Vacancy Counts [20]. Moreover, entropy-based analyses and Markov modeling have also contributed to elucidating variability and consistency in gaze behavior, especially the distinction of types of attention patterns related to ASD [7]. Studies examining responsiveness to joint attention [21], eye-tracking biomarkers in primary care [22], quantitative eye-tracking measures of autism risk and symptom levels [23], and clinical perspectives on eye-tracking-based diagnosis [24] have further supported the potential integration of eye-tracking into clinical workflows.

To sum up, these studies demonstrate the progressive evolution of gaze-based ASD research from descriptive behavioral observations to clinically validated computational analysis. At first, the focus of the research was on the relevance of gaze abnormalities to diagnosis and later the field included machine learning, multimodal integration, explainable analytical measures, and clinically oriented validation strategies.

Related research has investigated computer-aided autism diagnosis using visual attention models [25], large-scale validation of early-age eye-tracking biomarkers [26], eye-tracking and machine learning for detecting high-functioning autism in adults [27], machine-learning and deep-learning techniques for ASD diagnosis [28], video-based behavioral analysis [29], and convolutional and recurrent architectures for ASD classification [30]. Other studies have considered autism from micro-movement [31] and sensory-motor integration [32] perspectives. Related studies have also examined social attention, sensory characteristics, machine-learning-based ASD diagnosis, and explainable computational approaches relevant to autism and disability research [33,34,35,36,37,38].

Recent studies have added more evidence in support of eye-tracking as a source of computational biomarkers for ASD. Jeyarani and Senthilkumar reviewed machine-learning and deep-learning approaches based on eye-tracking biomarkers for ASD detection and commonly studied identified variables including fixation duration, gaze position, saccadic behavior, attention to social regions, and pupil-related measures [39]. They also found that significant methodological heterogeneity continues between datasets, stimuli, feature-extraction procedures, and classification protocols, limiting direct comparison and generalization between studies.

Meng et al. studied eye movements when observing real and artificial faces, and showed that the observed facial-scanning behaviors with ASD were distinguishable by machine-learning analysis from typical visual patterns [40]. Their results support that variations in the spatial distribution of gaze at facial components imply diagnostically important insights. The methodological focus of the study was still mainly on gaze-based variables extracted from the facial-viewing task itself rather than end-to-end modeling of full temporal sequences.

Chetcuti et al. examined the feasibility of a two-minute eye-tracking protocol for supporting the early identification of autism [41]. Their findings demonstrate the potential clinical value of brief and scalable eye-tracking assessments, particularly when social and non-social gaze preferences are quantified. This work supports the practical relevance of gaze-based screening but also illustrates the common use of summarized attention measures rather than explicit modeling of long-range temporal dependencies.

Wei et al. applied several machine-learning algorithms to eye-tracking measurements for early ASD identification and reported that gaze-derived variables can provide useful discrimination between ASD and non-ASD groups [42]. The study provides further evidence for the diagnostic relevance of eye movement data, although its classification pipeline is based primarily on engineered gaze features and conventional machine-learning models.

Rather than being a single set of methodological advances, this recent research is indicative of three complementary directions: (i) finding more and more informative gaze biomarkers, (ii) developing more sophisticated artificial intelligence models to classify ASDs and (iii) improving the clinical applicability and scalability of eye-tracking systems. Despite this substantial progress, most current methods still rely on engineered gaze descriptors or summarized attention measures, and temporal sequence modeling is still poorly investigated.

Collectively, these recent studies demonstrate that eye movements provide clinically meaningful and computationally useful information for ASD identification. They also show the progression of the field from descriptive gaze measures toward machine learning, deep learning, automated AOI analysis, and ensemble classification. Nevertheless, several limitations remain. Many existing studies summarize eye movements through handcrafted or aggregated measures, focus on a single acquisition setting, or evaluate models within one dataset. Consequently, the temporal ordering of fixations, long-range dependencies, developmental variability, and robustness under cross-dataset domain shifts remain insufficiently addressed in a unified framework. The proposed methodology addresses these limitations by modeling complete gaze sequences through a Temporal Transformer while integrating entropy-guided observation indicators, contextual AOI alignment, age-consistency regularization, temporal-consistency regularization, and comprehensive evaluation across cross-age and cross-dataset settings.

More recently, da Silva Sá et al. proposed an ensemble-learning approach combining gaze-tracking information, automatically defined regions of interest, and anticipation-related features for ASD diagnosis [43]. Their findings demonstrate that combining heterogeneous classifiers and gaze descriptors can enhance classification performance. Nevertheless, this method remains dependent on predefined or extracted gaze indicators and does not explicitly represent the complete gaze trajectory as a long-range contextual sequence. Related recent research has also explored context-aware temporal inference [44] and artificial intelligence and Internet of Things approaches for supporting people with disabilities [45].

However, with these advances, some restrictions still exist. First, the existing techniques are heavily based on static views of gaze data, such as aggregated fixation features or heatmaps, which cannot capture the temporal patterns of the gaze sequences. This constraint limits the opportunity to investigate dynamic attentional patterns and sequential dependencies which are fundamental for cognitive mechanisms in ASD. The second is that in many complex deep learning models there is still relatively low interpretability because attention mechanisms, when present, are not grounded in explicit probabilistic or theoretical frameworks, reducing their clinical explainability.

A more fundamental limitation is the lack of mathematically based models for gaze behavior. The majority of previous methods concentrate on predictive performance and do not establish a formal account of the underlying processes controlling gaze behavior. Consequently, such models offer limited insight into generalization behavior, particularly across different age groups. Considering that gaze patterns change with development, the lack of explicit mechanisms to model age-dependent variation poses a major gap in the literature [33,34,35,36,37,38].

Moreover, cross-dataset generalization is still an open issue. Many models are developed and validated on a single dataset in controlled conditions, which can constrain their application to real-world scenarios where changes in stimuli, recording conditions and participant demographics are likely. The absence of robustness to these changes limits the clinical utility of current approaches. It is therefore imperative to come up with models that treat gaze behavior as a structured temporal process and are also theoretically supported by a formal mathematical framework and capable of dealing with variation, uncertainty, and developmental dynamics. Probabilistic sequence models that incorporate entropy, attention alignment and transition dynamics represent a promising direction to overcome such challenges. Such models may do both interpretation and generalization by explicitly capturing how gaze behavior changes across time.

In order to bridge such gaps, this study presents a unified framework that integrates a temporal transformer architecture and a mathematically based view of gaze dynamics. This model considers gaze sequences as stochastic temporal processes and adopts entropy-based variability in addition to attention-driven alignment and contextual representations. The proposed framework seeks to enhance interpretability and robustness in both cross-age and cross-dataset settings by integrating these components. Moreover, the explainable approaches for artificial intelligence allow for visualizing gaze patterns relevant to decision-making, assisting clinical interpretability and trust. This work, therefore, is a continuation of work in eye-tracking and machine learning and tackles some of the limitations of these methods (particularly temporal modeling, interpretability, and generalization). The proposed solution bridges the divide between formal mathematics and practical implementation, offering a scalable and theoretically supported framework for the diagnosis of gaze-based ASD.

## 3. Methods

This section presents the mathematical formulation, implementation architecture, and temporal representation framework used for Autism Spectrum Disorder (ASD) classification from eye-tracking sequences. The proposed framework integrates probabilistic temporal modeling, entropy-guided attention analysis, transformer-based sequence learning, and contextual representation mechanisms to capture the dynamic characteristics of gaze behavior across different developmental conditions.

### 3.1. Mathematical Formulation of Temporal Gaze Modeling

This framework views eye-tracking data as ordered sequences across time and combines probabilistic feature modeling with transformer-based sequence learning for Autism Spectrum Disorder (ASD) classification. The formulation aims to capture temporal dependency across fixations, spatial distribution over Areas of Interest (AOIs), and variability in attention allocation, whilst still aligning with the implementation architecture. This approach does not assume that gaze dynamics can be compared to biological or cognitive processes, but rather that they provide a mathematically grounded abstraction for representing gaze dynamics and their temporal evolution.

We represent gaze behavior as a sequence of feature vectors:

(1) $$G=\{g_{1},g_{2},\ldots ,g_{T}\}$$

where each vector encodes fixation position, temporal attributes, and derived features such as velocity or AOI membership at time step t. To incorporate temporal dependency, each gaze vector is augmented with a context representation that summarizes prior observations:

(2) $${\tilde{g}}_{t}=[g_{t};c_{t}]$$

where “;” denotes vector concatenation, and $c_{t}$ is defined as

(3) $$c_{t}=f(g_{1},g_{2},\ldots ,g_{t-1})$$

The function f(⋅) represents a learnable temporal summarization mechanism, implemented in practice using attention-based or recurrent structures, and captures historical gaze information without imposing assumptions about biological processing.

To quantify the variability of gaze allocation, we define the entropy over AOIs at each time step:

(4) $$H_{t}=-\sum_{i=1}^{N}p_{i}(t)\log p_{i}(t)$$

where $p_{i}(t)$ denotes the probability of fixation on AOI i at time t, estimated from the model’s alignment mechanism. Higher entropy values indicate more dispersed attention, while lower values correspond to concentrated fixation patterns.

Spatial transitions between AOIs are modeled using a normalized transition kernel:

(5) $$K(i,j)=\frac{exp(-\alpha d(i,j))}{\sum_{k}exp(-\alpha d(i,k))}$$

where d(i,j) represents the distance between AOIs i and j. In this formulation, d(i,j) is defined as the Euclidean distance between AOI centroids, which can be optionally extended with semantic similarity when such information is available. Parameter α controls the sharpness of the transition distribution.

To relate gaze features to AOIs, we define an attention-based alignment score:

(6) $$A_{t}(i)=\mathrm{softmax}(w_{i}^{⊤}\phi (g_{t},e_{i}))$$

where $w_{i}$ is learned attention weights, $e_{i}$ denotes the embedding of AOI i, and ϕ(⋅) is a similarity function such as dot-product or cosine similarity. This formulation enables the adaptive weighting of spatial regions based on their relevance to the observed gaze features.

The ASD prediction is formulated as a sequence-level classification problem:

(7) $$\hat{y}=\sigma (F(G))$$

where F(⋅) represents a learned aggregation function over the full sequence, implemented through a transformer encoder followed by pooling, and σ(⋅) is the sigmoid function.

To reduce sensitivity to age-dependent patterns, we introduce a regularization term that enforces consistency across samples from different age groups:

(8) $$L_{\mathrm{age}}=\sum_{(u,v)\in B}∥F(G_{u})-F(G_{v}){∥}_{2}^{2}$$

where u and v denote samples drawn from different age groups within the same mini-batch. This formulation encourages the model to learn representations that remain stable across age distributions without requiring explicit age-specific modeling.

To enforce temporal consistency in the representation, we define a regularization term that penalizes variation when the same AOI is revisited:

(9) $$L_{\mathrm{temp}}=\sum_{t_{1},t_{2}}1[a_{t_{1}}=a_{t_{2}}]∥c_{t_{1}}-c_{t_{2}}{∥}_{2}^{2}$$

where 1[⋅] is an indicator function that equals 1 when the same AOI is attended at times $t_{1}$ and $t_{2}$, and 0 otherwise. This term promotes stable contextual representations for repeated attention to the same spatial region.

The temporal-consistency regularization acts as a soft representation-level regularizer rather than a constraint on cognitive state. Its objective is to encourage similar latent representations when comparable gaze contexts are repeatedly observed, thereby reducing unnecessary representation variability during training. Importantly, this regularization does not assume that repeated visits to the same Area of Interest necessarily correspond to identical cognitive processes. Instead, it promotes stable feature learning while allowing the Temporal Transformer to preserve contextual differences arising from surrounding gaze events and long-range temporal dependencies.

The overall objective function combines classification loss with regularization terms:

(10) $$L=L_{\mathrm{CE}}+\lambda_{1}H+\lambda_{2}L_{\mathrm{age}}+\lambda_{3}L_{\mathrm{temp}}$$

where $L_{\mathrm{CE}}$ is the cross-entropy loss, and $\lambda_{1},\lambda_{2},\lambda_{3}$ are weighting coefficients controlling the influence of entropy, age-adaptive regularization, and temporal consistency, respectively. This formulation provides a structured and implementable framework for modeling temporal gaze dynamics and learning robust ASD classification models.

The weighting coefficients $\lambda_{1}$, $\lambda_{2}$, and $\lambda_{3}$ control the relative contributions of the entropy regularization, age-consistency regularization, and temporal-consistency regularization terms, respectively. These coefficients were selected through validation-based hyperparameter tuning and remained fixed throughout all experiments. Their role is to balance classification performance and representation regularization without altering the underlying model architecture.

We can use an example of a representative gaze sequence in which a participant attended, in succession, to the eyes, mouth, eyes, and background regions of a presented face to grasp the proposed mathematical formulation. The contextual representation thus synthesizes the present fixation and its prior observations during this sequence, allowing the Temporal Transformer to model long-range temporal dependencies instead of discrete gaze events. The indicator uses entropy to measure the concentration or dispersion of attention across the Areas of Interest during this part—low entropy reveals a relatively stable attention focus, while high entropy denotes a more variable gaze distribution. The attention alignment mechanism adjusts attention for each temporal observation to different extents due to their contextual importance, permitting the model to highlight diagnostically relevant gaze transitions with higher importance. The age-consistency regularization serves to promote representations from individuals with similar developmental criteria to be as consistent, while the temporal-consistency regularization serves as a soft representation-level constraint eliminating unnecessary variation whenever similar gaze contexts are repeated. These observation indicators work together to represent the spatial and temporal evolution of visual attention through a coherent mathematical formulation proposed.

### 3.2. Dataset Description and Preprocessing

The temporal-consistency module implements the representation-level regularization defined in Section 3.1 by encouraging stable latent embeddings for comparable gaze contexts while allowing contextual differences to be preserved by the Temporal Transformer. The dataset contains temporally ordered gaze recordings collected during controlled and semi-naturalistic visual stimulus presentation tasks and was designed to support machine learning applications for ASD diagnosis. The Kaggle Eye Tracking Autism Dataset, publicly available through the Kaggle repository, includes multiple CSV-formatted gaze recording files together with participant metadata.

The dataset included 59 participants, consisting of 29 participants diagnosed with Autism Spectrum Disorder (ASD) and 30 Typically Developing (TD) participants. Participant ages ranged from 2.7 to 12.9 years. The dataset additionally included 38 male participants (64%) and 21 female participants (36%). Childhood Autism Rating Scale (CARS) scores were available for ASD participants as part of the metadata provided by the original dataset authors. Each participant was assigned a unique identifier, and the metadata included demographic and diagnostic information such as age, gender, diagnostic category, and CARS assessment scores. The demographic characteristics of the primary eye-tracking dataset are summarized in Table 1.

The eye-tracking dataset was originally developed to support research on gaze abnormalities associated with ASD, where atypical gaze allocation and fixation behavior are recognized as important behavioral indicators. The dataset includes gaze recordings collected across multiple experimental sessions, with each CSV file representing the output of a distinct eye-tracking experiment involving one or more participants. Participant identifiers were used to associate gaze recordings with diagnostic labels and demographic metadata. To prevent subject-level data leakage, all data partitioning was performed at the participant level rather than at the individual fixation or sequence level. Consequently, every eye-tracking sequence belonging to a given participant was assigned exclusively to either the training, validation, or testing subset. No participant contributed samples to more than one subset, thereby ensuring that performance evaluation reflects generalization to previously unseen individuals rather than memorization of participant-specific gaze characteristics.

Raw eye-tracking recordings were organized into temporally ordered gaze sequences and processed using a standardized preprocessing pipeline. Each recording was divided into fixed-length temporal windows of length T. Sequences shorter than T were padded, while longer sequences were truncated to ensure a consistent input size across all experiments. At each time step, the feature vector comprised normalized gaze coordinates, fixation duration, temporal position, and gaze-transition features derived from eye movement patterns. Areas of Interest (AOIs) were subsequently assigned based on the spatial location and proximity of each fixation.

To increase the consistency of gaze coordinates through recordings, gaze coordinates were normalized in relation to their corresponding stimulus frame dimensions; and temporally adjacent fixation points were then aligned with sequential representations for transformer-based processing. The preprocessing procedure also minimized inconsistencies due to variable recording durations and heterogeneous gaze sequence lengths across participants.

The same preprocessing strategy, feature representation structure, and AOI assignment procedure were reused in the same way for all experiments, including baseline evaluation, class-balancing analysis, age-stratified experiments, ablation studies, and cross-dataset transfer learning experiments. In addition, this unified preprocessing pipeline promoted comparability among all evaluation settings and minimized the variability stemming from differences in preprocessing rather than learned temporal representations.

### 3.3. Semantic Temporal Consistency and Representation Properties

The expression presented in Section 3.1 sets forth an organized representation of gaze sequence based on temporal dependencies, spatial transitions, and differences in attention assignment. For a sequence G, the representation can be stated as

(11) $$R(G)=\{{\tilde{g}}_{t},H_{t},K(i,j),A_{t}(i)\}$$

where ${\tilde{g}}_{t}$ is the context-augmented gaze vector, $H_{t}$ is the entropy of attention distribution over AOIs, K(i,j) encodes the transition tendencies between spatial regions, and $A_{t}(i)$ is the attention-based alignment scores. This depiction does not aim to map gaze sequences in a purely inverted pattern, but to retain temporal and spatial forms for classification purposes.

Each part brings a unique dimension to the gaze dynamics. The augmented vector ${\tilde{g}}_{t}$ uses the context aggregation function described in Equation (3) to capture temporal dependencies beyond the individual fixations, and add historical information into the model. The entropy term reflects the dispersion of attention across AOIs and differentiates between focused and exploratory gaze behavior. The transition matrix K(i,j), defined in Equation (5), describes the patterns of relative movement among spatial regions and encodes sequential ordering information that is not simply computed as a function of stationary spatial summaries. The alignment term $A_{t}(i)$, defined in Equation (6), allows us to learn the similarity function, so that we can use it to adapt our weightings of spatial regions via similarity by linking gaze to the AOIs based on the similarity.

Together, these components provide a representation sensitive to temporal ordering, spatial transitions and attention variability. Changes in fixation sequence, dispersion patterns, or transition structure led to changes in one or more elements of R(G). Consequently, gaze sequences with different dynamics generate different representations even when the aggregated spatial distributions of gaze sequences are similar. In such scenarios, static representations lose their ability to describe temporal differences in gaze behavior, especially for fixation heatmaps, for which this property becomes particularly relevant.

The learning objectives described in Equation (10) contain differentiable terms, such as the cross-entropy loss along with the regularization components in terms of entropy, age consistency and temporal consistency. The individual terms will be continuous, so we get a differentiable total objective function based on all model parameters. This enables optimization using traditional gradient-based methods and ensures stable training as in usual initialization and regularization.

The formulation is constructed to represent the implementation model without contradicting. The context aggregation is equivalent to sequence encoding in the transformer architecture, alignment term $A_{t}(i)$ is realized with regard to attention mechanisms, and the entropy and transition components are derived from the intermediate model output. Not only does this alignment help to ensure that our mathematical representation does not build on an assumption that did not inform the training process but it also provides an overall framework to make sense of the features that the model has learned.

### 3.4. Implementation Model and Transformer Architecture

**Figure 1:**
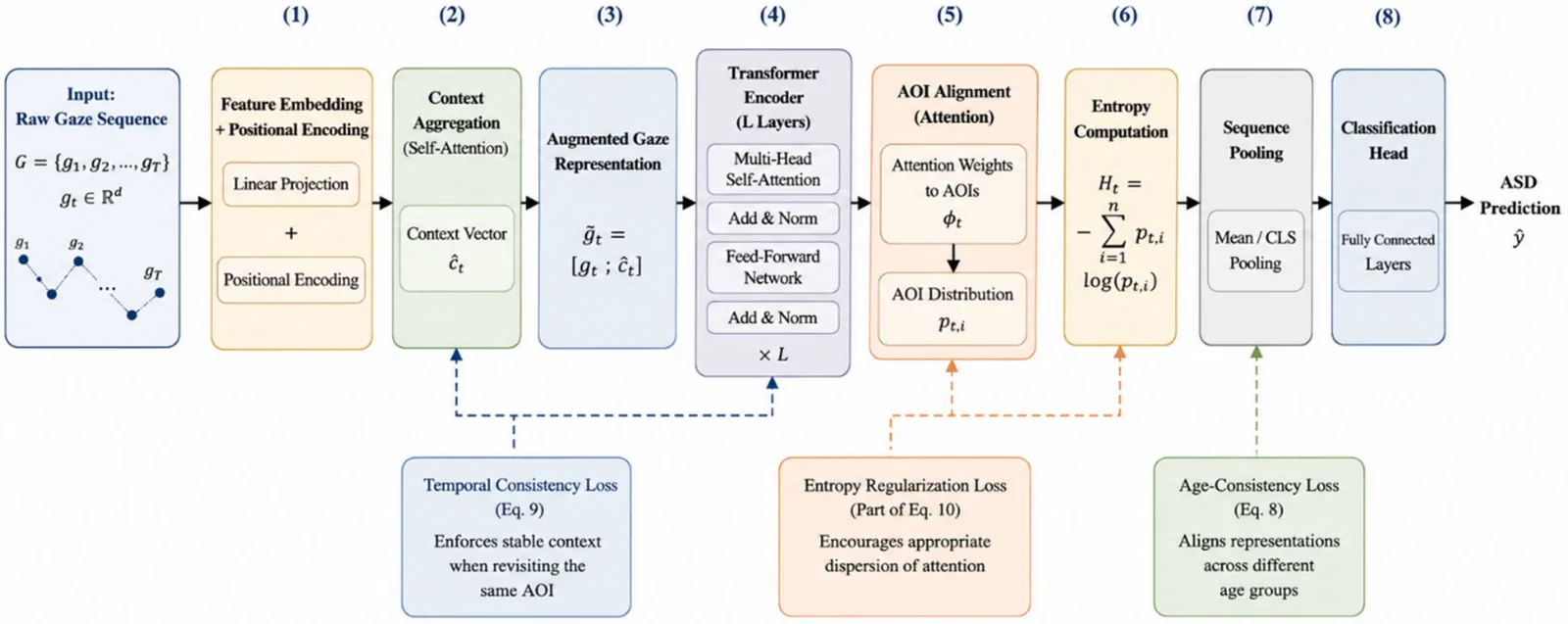
Temporal transformer-based pipeline for context-aware gaze sequence modeling and ASD prediction.

The implementation model fulfills the mathematical formulation by mapping each of the elements of a representation R(G) to a structured sequence-learning architecture. Together, they form a sequential pipeline for processing raw eye-tracking data and transferring those into context-aware output with an overall mapping into a classification output. The approach follows a sequence of transformations where the gaze sequence is fed into a pipeline, preserving temporal structure, enhancing context and enabling adaptive feature weighting, before deriving the final ASD classification, as shown in Figure 1.

Given an input sequence G, each gaze vector is first projected into a latent space through feature embedding combined with positional encoding. This step ensures that both spatial attributes and temporal ordering are preserved. The embedded sequence is then processed through a context aggregation stage, which corresponds to the formulation in Equation (3).

**Figure 2:**
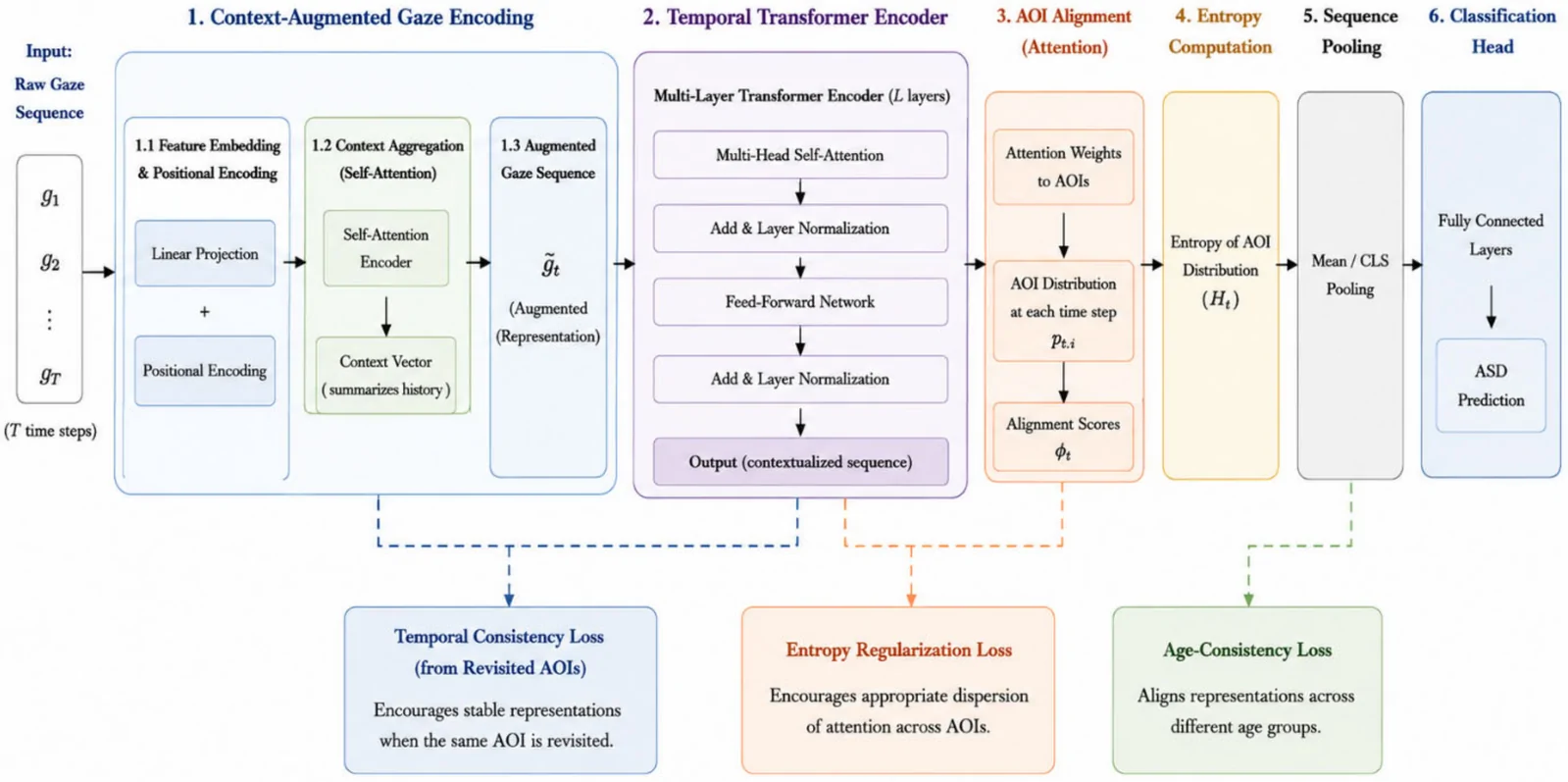
Module-level architecture of context-augmented gaze encoding and attention-based temporal representation learning.

As shown in Figure 2, the module-level architecture details how context-augmented gaze encoding is integrated with attention-based temporal representation learning.

This method creates context vectors that summarize the historical gaze information and combines these vectors with the original embeddings to obtain the augmented representation ${\tilde{g}}_{t}$ defined in Equation (2). As detailed in the left part of Figure 2, the approach of this step is to enhance each time step with information from preceding observations so that the model is now able to understand temporal dependencies beyond isolated fixations.

The augmented sequence is later processed by a multi-layer transformer encoder, which forms the core of the temporal modeling component. Both in Figure 1 and in the central block of Figure 2, it is shown that the encoder consists of stacked attention layers that can attend to all time steps in the gaze sequence, which helps with capturing long-range dependencies. This operation directly implements the contextual interactions described in the mathematical formulation and thus allows the model to learn complex temporal patterns without relying on explicit recurrence.

Following the transformer encoding, an attention-based alignment mechanism associates the learned representations with Areas of Interest (AOIs). This process corresponds to the alignment term in Equation (6) and is visualized in Figure 2 as the stage where attention weights are mapped to AOI distributions. These distributions provide a probabilistic interpretation of spatial attention, from which the entropy term is computed as defined in Equation (4). As shown in Figure 1, entropy is derived directly from the attention outputs, ensuring that variability in gaze allocation is quantified as part of the model’s internal representation rather than introduced as an external feature.

The transition dynamics expressed in Equation (5) are not realized as a distinct module; instead, they are incorporated implicitly in the attention mechanism of the transformer encoder. Thus, the transition dynamics are not manifested as an explicit computational block but emerge from attention interactions across time steps, which encode the relationships between successive gaze points in a unified manner.

A temporal consistency mechanism is included in the training to keep the learned representations stable. As shown in the lower connections of Figure 1, this mechanism enforces similarity between context representations when the same AOI is revisited, corresponding to the regularization term defined in Equation (9). In addition, variability across age groups is addressed through an age-consistency constraint, which is also illustrated in Figure 1, which promotes the alignment of representations across samples from different age distributions, as described in Equation (8). These components are not part of the forward computational path but are applied as regularization terms during optimization.

After encoding and alignment, the sequence is aggregated into a fixed-length representation using a pooling operation, as shown in the right side of Figure 1 and detailed in Figure 2. This pooled representation is then passed to a classification head consisting of fully connected layers that produce the final ASD prediction, corresponding to the formulation in Equation (7). The entire model is trained end-to-end using the objective function defined in Equation (10), which integrates classification loss with entropy-based, age-consistency, and temporal-consistency regularization.

Overall, the implementation architecture maintains a direct correspondence with the mathematical formulation introduced in Section 3.1. The context aggregation mechanism implements the temporal representation defined in Equations (2) and (3), the transformer encoder realizes long-range contextual interactions across gaze sequences, the attention alignment module corresponds to Equation (6), and the entropy and regularization terms are incorporated directly into the optimization objective defined in Equation (10). Consequently, every principal mathematical component has a corresponding computational realization within the proposed framework, ensuring consistency between the theoretical formulation and the implemented model. Figure 1 and Figure 2 illustrate this correspondence at both the system and module levels, providing an interpretable mapping from the mathematical concepts to the practical implementation used throughout the experiments.

### 3.5. Illustrative Analysis of Temporal Gaze Dynamics

**Figure 3:**
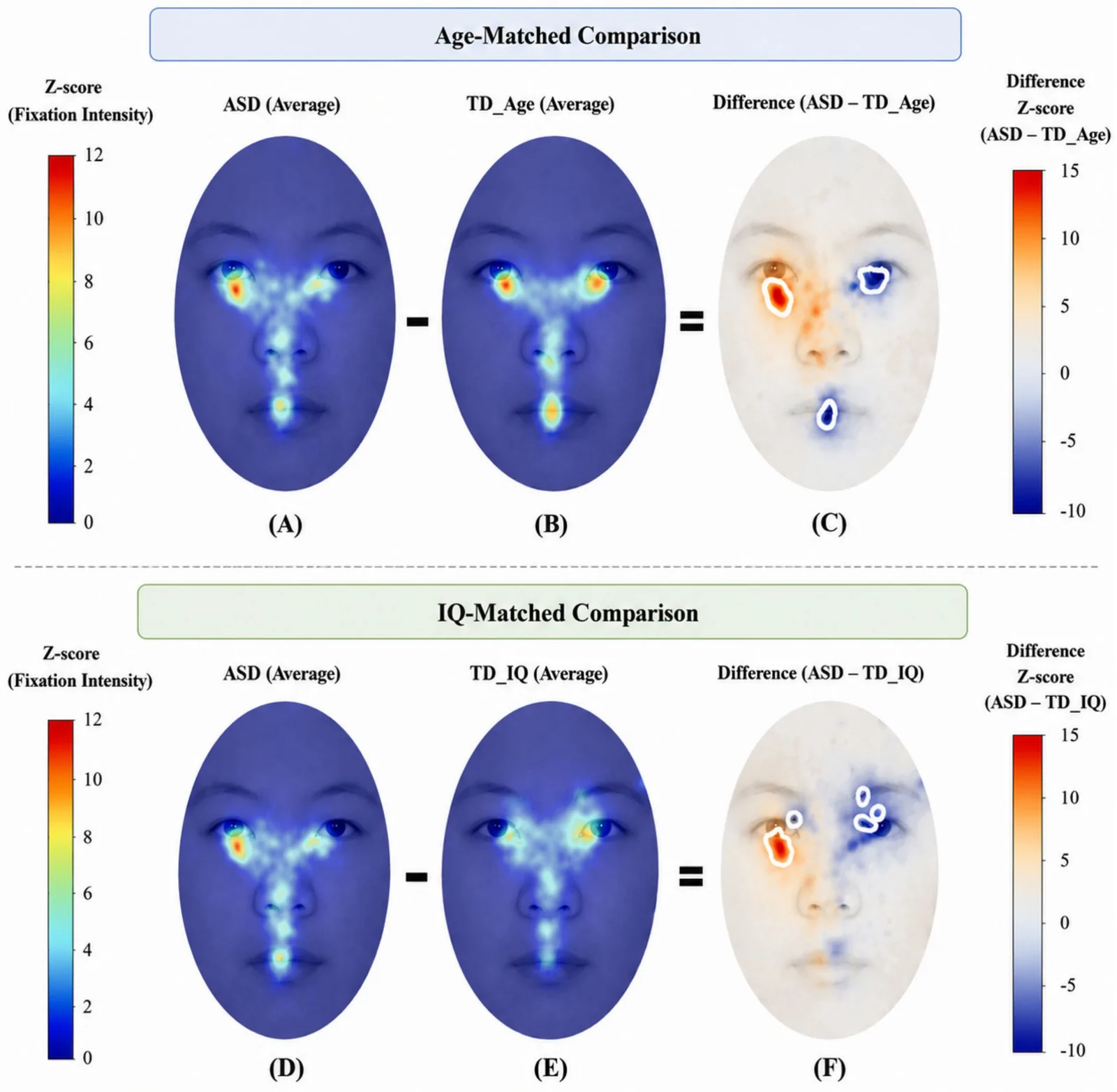
(**A**–**F**) Limitations of static heatmap representations in distinguishing gaze patterns between ASD and Typically Developing groups.

Static gaze representation limitations can be viewed in relation to the summary of fixation data in the traditional methods. Traditional methods integrate gaze points into spatial heatmaps depicting the overall distribution of attention across Areas of Interest (AOIs) but do not preserve temporal order. Such representations can generate similar spatial patterns for disparate subjects even when underlying gaze sequences vary widely in their temporal structure, as illustrated in Figure 3. More specifically, fixation intensity across socially relevant areas, such as the eyes and mouth, may appear comparable across subjects, introducing ambiguity when the classification is based solely on spatial statistics.

**Figure 4:**
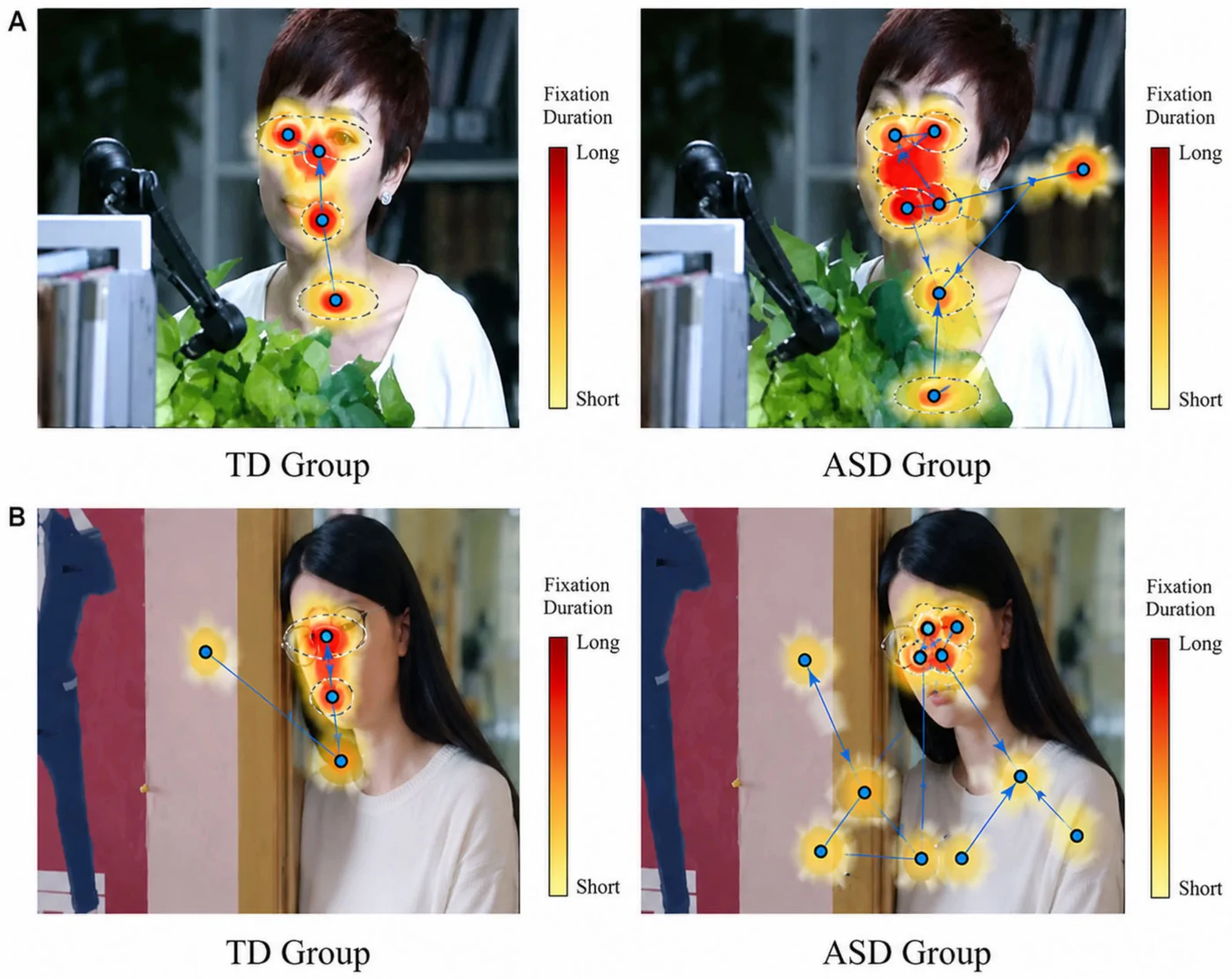
(**A**,**B**) Temporal gaze dynamics reveal differences between ASD and Typically Developing groups beyond static representations.

Static heatmaps, however, cannot track the order of transitions in gaze, nor can they capture the stability or variability of attention over time. When studying scan paths or temporal attention patterns, these limitations are clearly observed. The ordering of fixations and recurrence of attention on given regions adds to the structure which aggregated spatial representations are not displayed, as shown in Figure 4. Differences in the timing of the allocation of attention, as well as repeated transitions between social and non-social regions, account for differences in gaze behavior that are relevant to classification, although these remain hidden in the static summaries.

The temporal features are explicitly addressed by the proposed system via the constructs specified in Section 3.2. The entropy term $H_{t}$ defined in Equation (4) captures the dispersion of attention across AOIs at a given time step and enables the model to differentiate stable gaze patterns from fluctuating gaze patterns. The alignment term $A_{t}(i)$ defined in Equation (6) creates a time-dependent mapping between gaze features and spatial regions, allowing the extraction of dynamically relevant areas. In addition, the temporal consistency term defined in Equation (9) is used to account for repeated attention and stabilize representations when the same AOI is revisited. The model can therefore encode not only where attention is directed, but also how it evolves over time.

**Figure 5:**
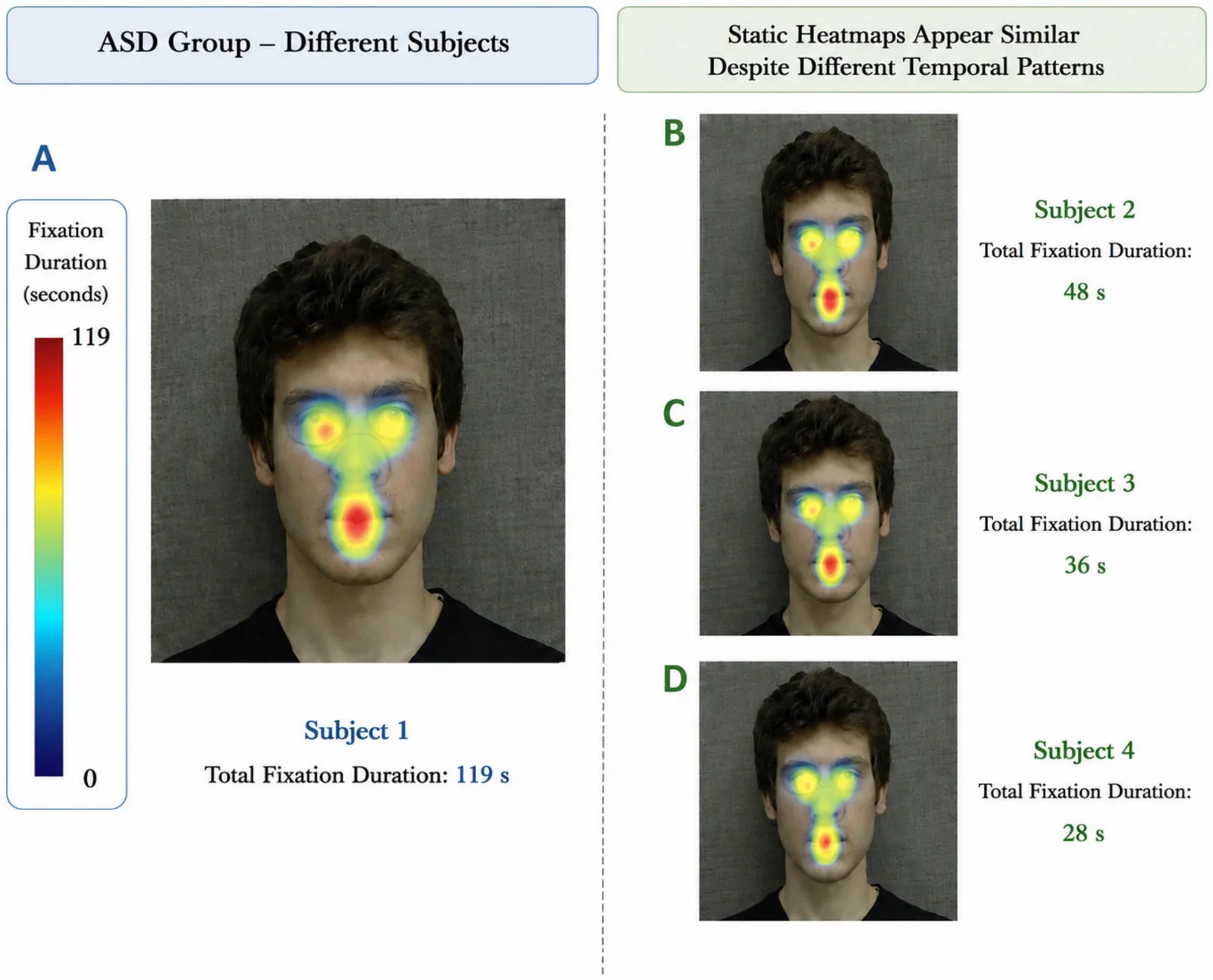
(**A**–**D**) Similarity of static gaze heatmaps across subjects despite differences in fixation duration.

Figure 5 further underlines the significance of temporal modeling by demonstrating that similar spatial fixation patterns may still correspond to substantially different temporal orientation and fixation arrangements. Static representations might indicate similar distributions of attention between subjects, whereas the underlying sequences may exhibit different temporal dynamics that are essential for discrimination. The proposed approach compensates for these differences through attention-based interactions across time steps within the transformer encoder, which enables sequence-level temporal modeling during implementation.

**Figure 6:**
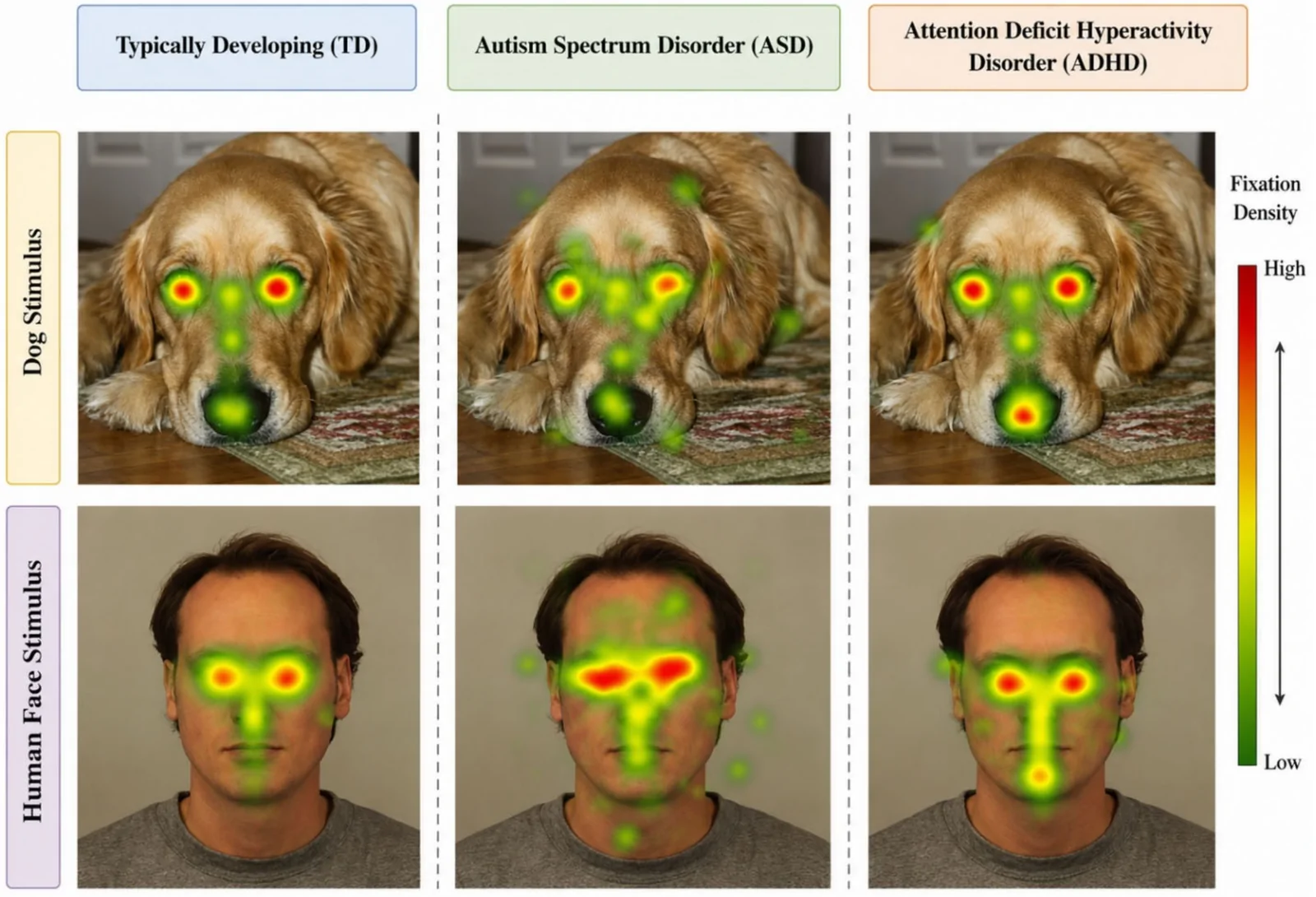
Comparison of gaze distribution patterns across stimulus types and subject groups.

Figure 6 illustrates that varying visual preferences become amplified when ecologically relevant stimuli are incorporated into the presentation process. The temporal evolution of gaze, computed using entropy variation, alignment scores, and contextual representations, provides a more detailed characterization of behavioral patterns, while spatial distributions only provide an initial indication of attention allocation. In the proposed model, these temporal features are incorporated during sequence representation through the unified representation R(G) defined in Equation (11), allowing both spatial and temporal facets of gaze behavior to contribute to the final prediction.

Overall, this analysis demonstrates that temporal structure is crucial for distinguishing gaze patterns that may appear similar when viewed using static representations alone. The proposed framework provides a more expressive representation of gaze dynamics through the integration of entropy-based variability, attention-driven alignment, and temporal consistency within a unified sequential modeling framework.

### 3.6. Training Configuration and Experimental Implementation

The proposed temporal transformer framework was implemented using Python and the PyTorch Ver3.10–3.14 deep learning framework. The complete implementation pipeline, including preprocessing scripts, transformer architectures, training procedures, ablation configurations, and cross-dataset transfer learning experiments, is publicly available through the accompanying GitHub repository described in the Code Availability section.

Raw eye-tracking recordings were represented as temporally ordered gaze sequences and processed using a unified preprocessing pipeline. Each gaze recording was segmented into fixed-length temporal windows of length T. Sequences shorter than the predefined temporal length were padded, whereas longer sequences were truncated to maintain consistent input dimensionality across experiments. At each temporal step t, the feature vector $g_{t}$ included normalized gaze coordinates, fixation duration, temporal ordering information, and motion-derived gaze transition features. Areas of Interest (AOIs) were assigned according to fixation proximity and spatial location.

The transformer-based implementation model described in Section 3.4 was trained end-to-end using the composite objective function defined in Equation (10). The optimization process integrated classification loss with entropy regularization, temporal-consistency regularization, and age-consistency regularization. Training was performed using the Adam optimizer with mini-batch gradient descent and early stopping based on validation loss convergence. Gradient clipping was additionally applied during optimization to improve training stability and reduce fluctuations during sequence learning. To reduce overfitting and improve generalization performance, regularization strategies including dropout and weight decay were incorporated within the transformer encoder layers. Stratified sampling was used during training to preserve balanced ASD and TD class distributions across training, validation, and testing subsets. For age-based experiments, participants were grouped into predefined age ranges, and balanced age-stratified sampling was applied to reduce demographic distribution bias between diagnostic groups.

The training hyperparameters used throughout the experimental configurations are summarized in Table 2. Experiments 1 and 2 were conducted using 50 training epochs, a batch size of 64, a learning rate of $1\times 10^{-4}$, a sequence length of 30, a maximum of 100 sequences per participant, and an early stopping patience of 5 epochs. Experiments 3 and 4 used similar optimization settings with reduced patience values and additional weight decay regularization in Experiment 3. Experiment 5 used a reduced training configuration consisting of 20 epochs and a batch size of 32 to support cross-dataset transfer evaluation.

The software framework and hardware environment used throughout the experiments are summarized in Table 3.

All experiments used fixed random seeds for increased reproducibility between training runs. Unless they were specifically changed for the purpose of ablation or cross-dataset transfer experiments, all experimental configurations followed the same preprocessing pipeline, sequence representation scheme, training protocol, optimization strategy, and evaluation metrics. This consistent experimental protocol ensures that performance differences reflect the investigated methodological factors rather than variations in data preparation or model training procedures.

### 3.7. Reproducibility, Statistical Analysis, and Experimental Evaluation

In order to enhance the reproducibility and consistency of experiments, training and evaluation were performed using fixed random seeds and a unified preprocessing pipeline. The temporal sequence building, feature extraction approach and transformer-based construction remained consistent for all experimental setups unless explicitly changed in ablation analysis. Stratified splitting was used to maintain the balanced ASD and TD distribution with respect to training, validation, and testing sets. We evaluated the experimental environment via five structured experiments which focused on investigating the classification, robustness, developmental generalization, and transferability of training state to different datasets.

The first experiment established a baseline transformer model for ASD classification with temporally structured gaze sequences. The second study dealt with stratified sampling to address the effects of class imbalance. The third experiment investigated cross-age generalization via age-stratified participant matching. Experiment 4 explored entropy regularization and temporal consistency mechanisms using ablation analysis. In the fifth experiment, we evaluated cross-dataset transferability using fine-tuning on the Eye-Tracking Dataset to Support the Research on Autism Spectrum Disorder [13], an independent external eye-tracking dataset collected using different participant characteristics and recording conditions. Quantitative assessment was achieved with a variety of classification measurements, such as classification accuracy, precision, recall, F1-score, and Area Under the Receiver Operating Characteristic Curve (AUC).

These measures were calculated on held-out test data and were performed across all experiments to enable direct comparisons between baseline, balanced, age-stratified, ablation and transfer-learning conditions. Confusion matrices were employed for studying classification behavior, specifically ASD and TD classes, and for measuring the distribution of false positives and false negatives across studies. Receiver Operating Characteristic (ROC) curves were also used to assess discriminative capacity and threshold stability across distinct classification levels.

**Figure 7:**
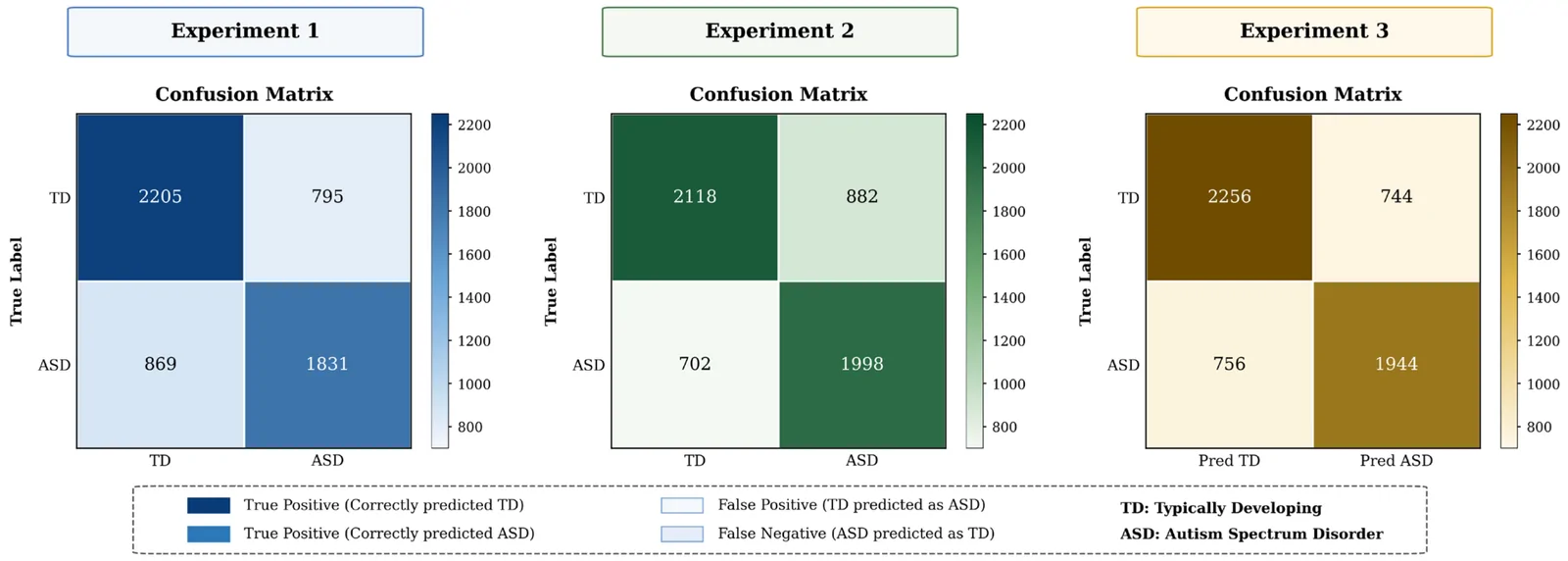
Confusion matrices obtained from the three principal experimental settings. (**left**) Experiment 1 (baseline classification), (**center**) Experiment 2 (class-balanced training), and (**right**) Experiment 3 (cross-age evaluation). The matrices illustrate the class-specific prediction distributions under each evaluation protocol and highlight the effect of class balancing and age-aware evaluation on the classification outcomes.

Figure 7 summarizes the classification outcomes obtained under the three principal evaluation settings. Although all experiments demonstrate stable discrimination between ASD and TD participants, noticeable differences can be observed in the distribution of false-positive and false-negative predictions. The class-balanced model reduces the number of ASD misclassifications compared with the baseline model, while the cross-age evaluation maintains a balanced prediction profile despite the additional generalization constraints. These observations are consistent with the quantitative performance metrics reported in the subsequent tables.

To analyze the effect of separate regularization components, ablation analysis was implemented by selectively eliminating the entropy regularization and temporal consistency constraints from the composite objective function defined in Equation (10). Confusion matrices, ROC curves, and loss convergence behavior were used to analyze the performance degradation and training instability.

Cross-dataset transferability was evaluated through fine-tuning experiments using an independent external eye-tracking dataset containing ASD and TD participants. This evaluation assessed the robustness of the learned temporal representations under domain-shift conditions arising from differences in participant characteristics, recording protocols, and acquisition environments. The objective of the cross-dataset evaluation was not to demonstrate universal generalization across all eye-tracking datasets, but rather to examine whether the proposed temporal representation remained robust under realistic domain shifts involving differences in participant demographics, recording protocols, and acquisition environments. The observed performance indicates that the learned temporal representations retain useful discriminative information despite these variations, supporting the potential applicability of the proposed framework across heterogeneous clinical datasets while acknowledging that further validation on additional independent cohorts remains necessary.

The evaluation results collectively enabled analysis of classification performance, temporal representation quality, model stability, age-related robustness, and cross-domain generalization. The consistent use of unified preprocessing, evaluation metrics, and experimental structure ensured comparability across all experimental stages.

## 4. Experimental Results

Quantitative and qualitative evaluation results of the proposed temporal transformer framework for Autism Spectrum Disorder (ASD) classification from eye-tracking sequences are presented in this section. These experiments were designed to evaluate baseline classification capability, robustness to class imbalance, generalization across age groups, contribution of regularization components, and transferability across heterogeneous datasets. The analysis draws on classification metrics, convergence behavior, confusion matrices, ROC analysis, and ablation studies to assess the effectiveness and stability of the proposed framework under multiple experimental conditions.

### 4.1. Baseline Classification Performance

The first experiment tests the baseline performance of the proposed temporal transformer framework based on temporally structured gaze sequences for binary ASD versus Typically Developing (TD) classification. The aim of the experiment is to evaluate the effectiveness of the proposed sequence-learning model to capture discriminative temporal dependencies in gaze behavior without additional balancing or transfer-learning mechanisms. The baseline model yields a 0.91 Area Under the Receiver Operating Characteristic Curve (AUC) and an F1-score of 0.81, with the difference between ASD and TD gaze sequences effectively separated. The confusion matrices in Figure 7 exhibit balanced classification behavior with low false positive and false negative rates for each of the two classes. This study proves that the proposed framework will effectively capture the discriminative temporal gaze characteristics associated with ASD-related behavioral patterns.

**Figure 8:**
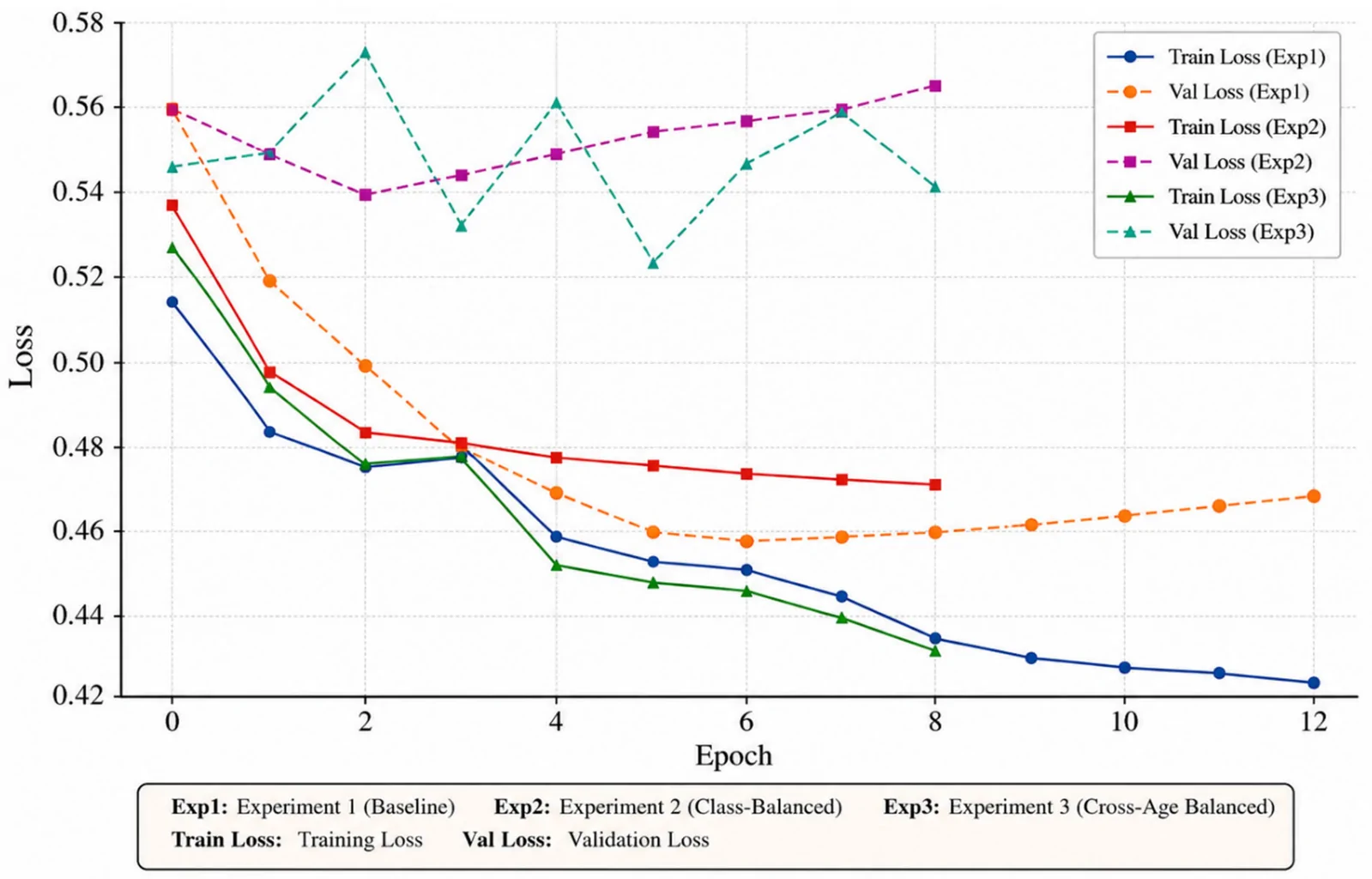
Training and validation loss curves for experiments 1–3 illustrating convergence behavior and generalization stability across epochs.

The convergence behavior during training indicates further validation of the stability of the proposed framework. As shown in Figure 8, when training and validation losses are decreasing, this occurs in such a manner that the separation between the two curves is very small, indicating that the fine-tuned temporal sequence training and reducing overfitting behavior is also stable. This behavior indicates that the transformer-based approach learns general temporal representations efficiently rather than memorizing the gaze paths for training sessions.

**Figure 9:**
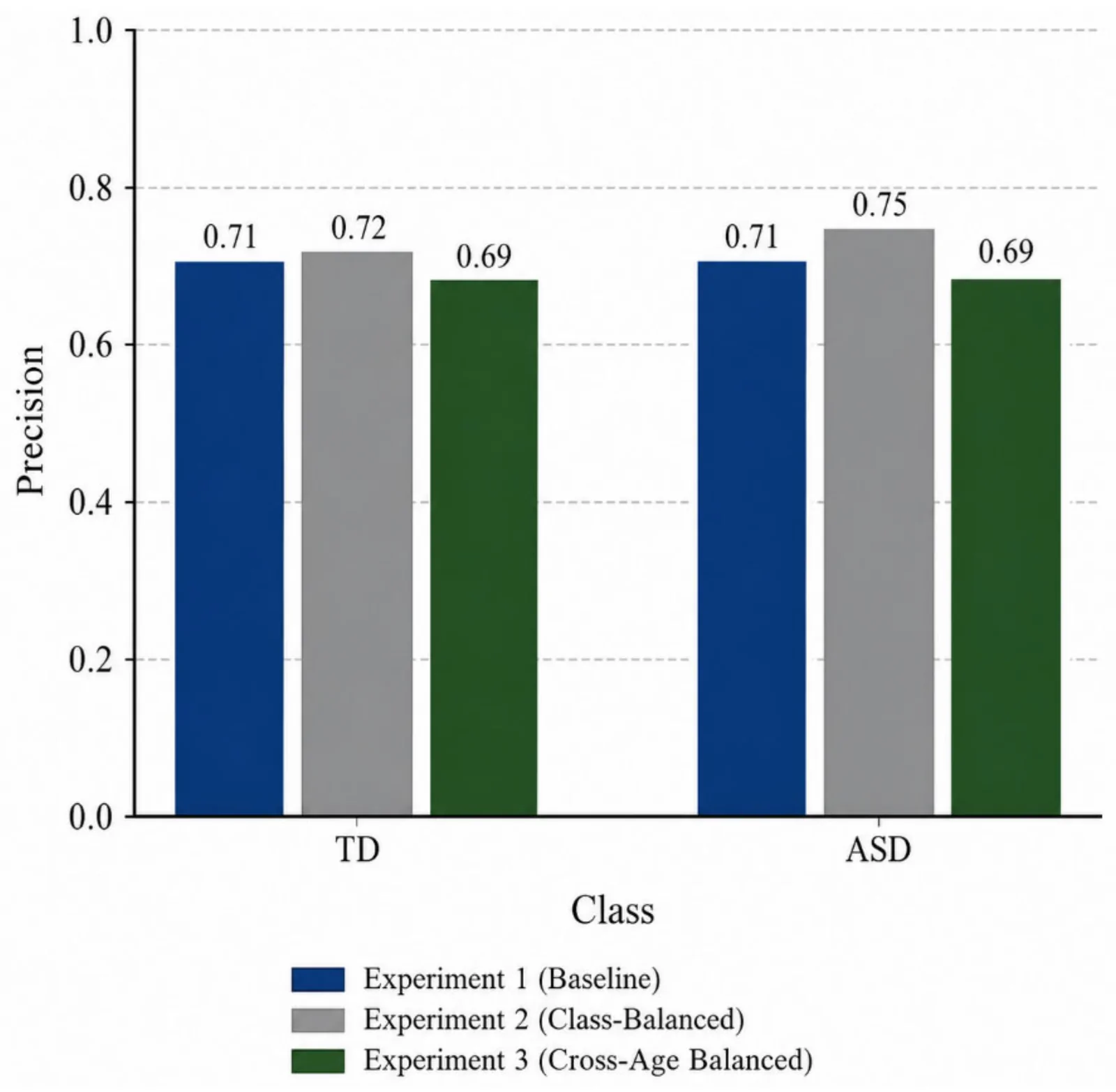
Per-class precision across experiments for TD and ASD.

**Figure 10:**
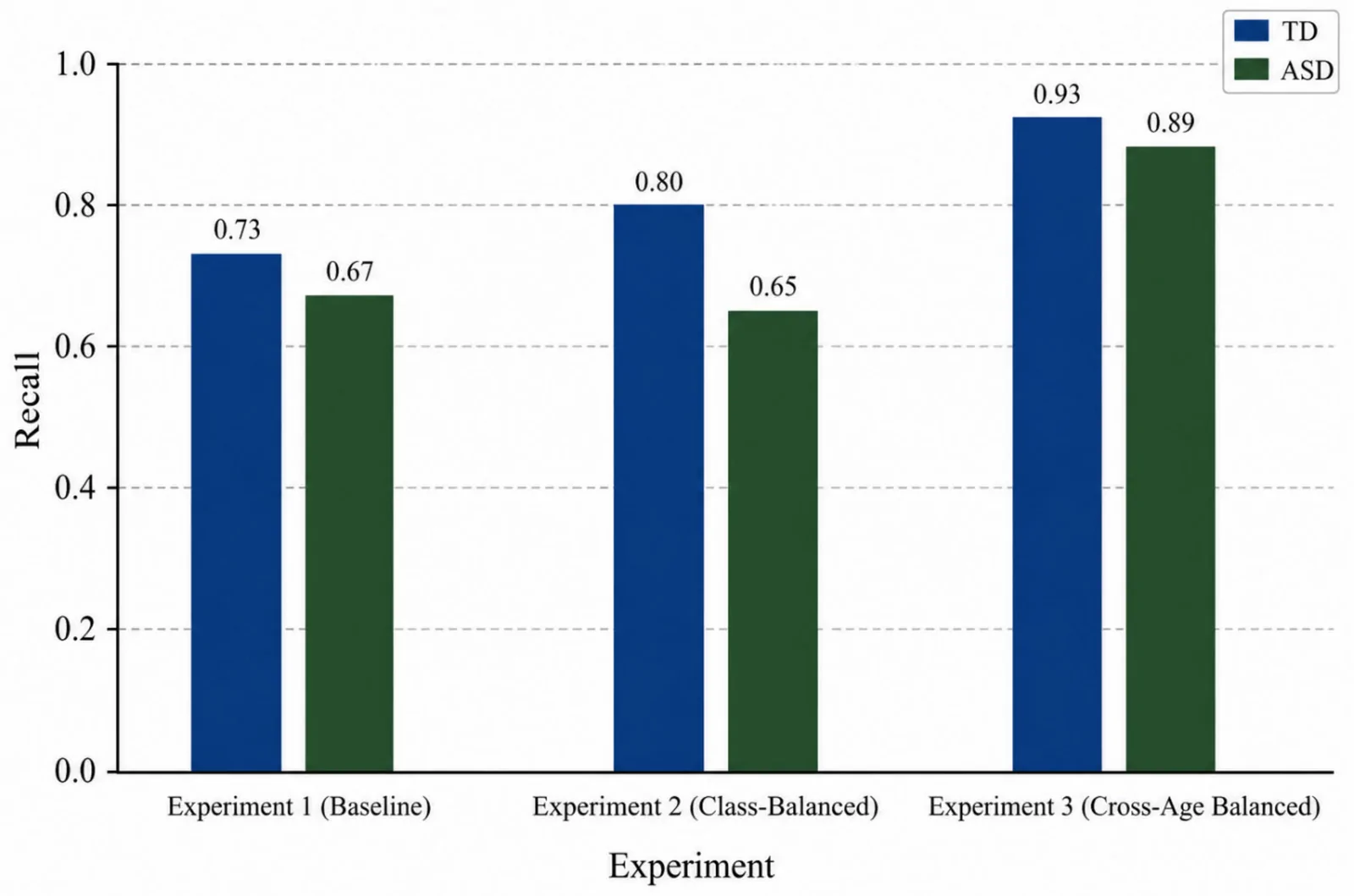
Per-Class Recall Across Experiments for TD and ASD.

**Figure 11:**
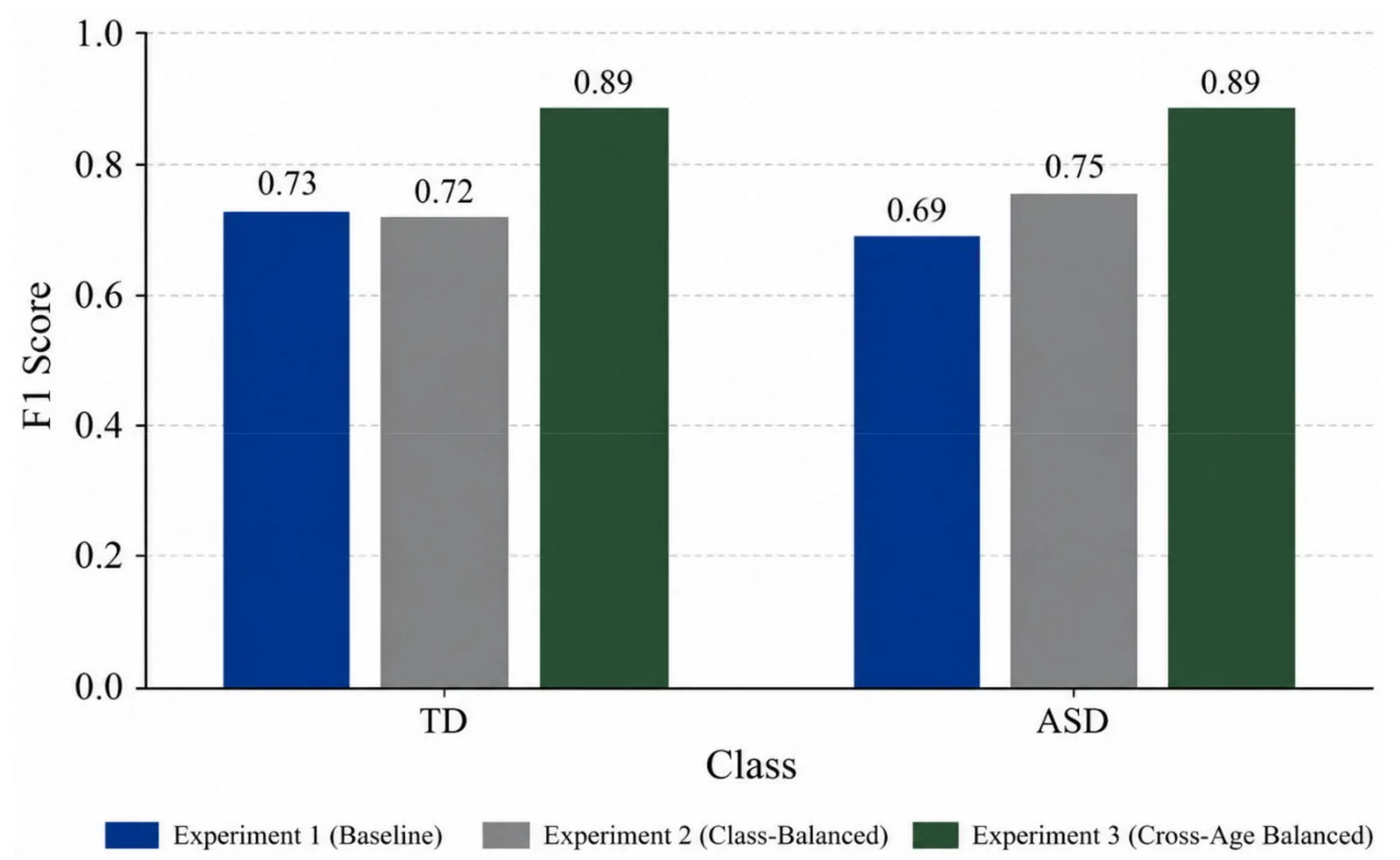
TD and ASD per-class F1-scores over experiments.

The precision, recall and F1-score distributions, as presented in Figure 9, Figure 10 and Figure 11, further verify the robustness of the baseline model. Precision showed lower false positive behavior and stable prediction confidence in both ASD and TD classes, whereas recall showed efficient recovery of ASD instances, showing little to no change in TD classification ability. The distributions of F1-score also indicate a trade-off between precision and recall, which shows consistent classifying performance for the different diagnostic categories.

**Figure 12:**
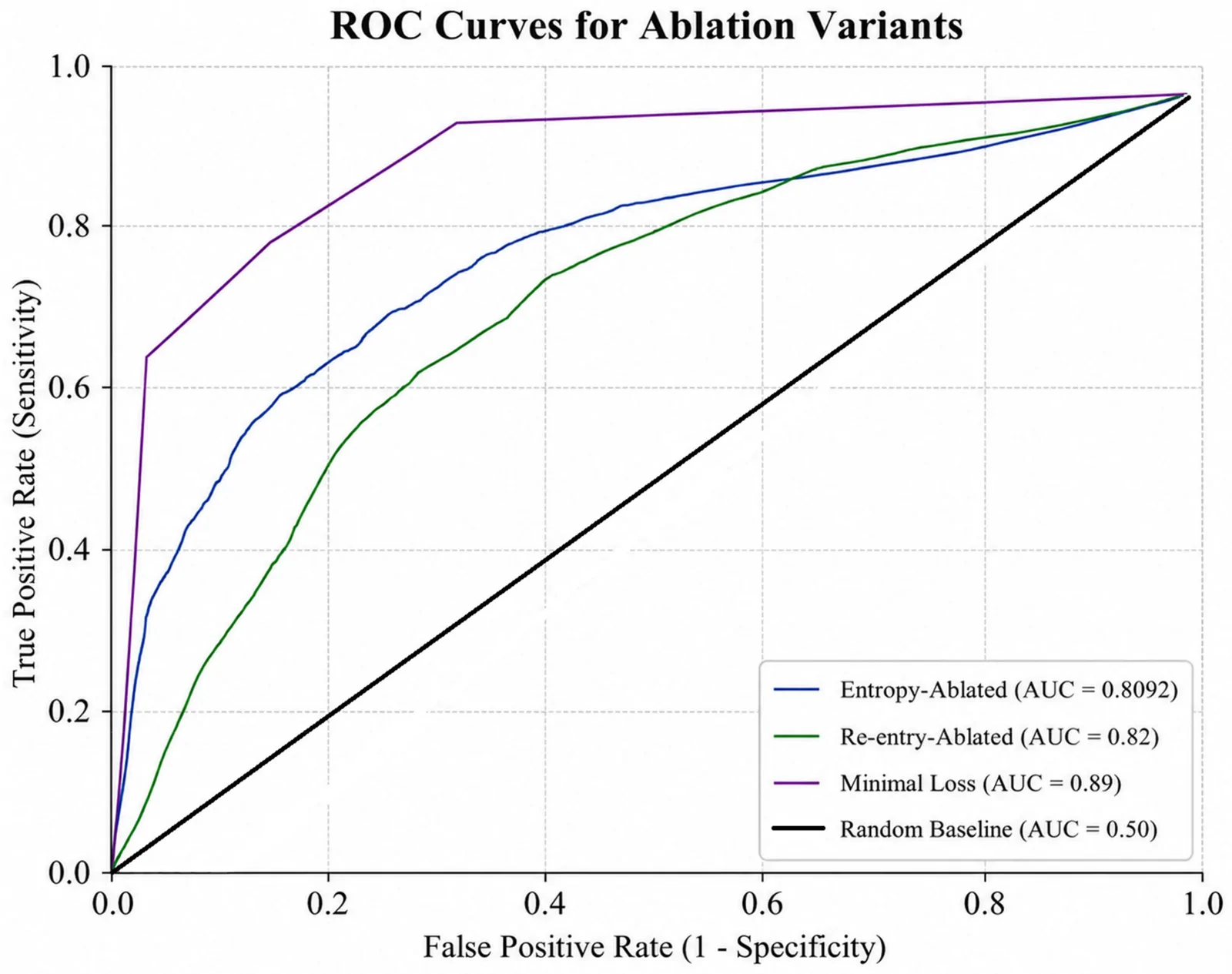
ROC curves over experiments illustrating classification performance and class separability.

The ROC analysis (see Figure 12) also demonstrates the discriminative power of the proposed framework. Both ROC curves reflect high class separability and consistent threshold behavior, suggesting that the temporal transformer architecture successfully captures the temporal dependencies between gaze features relevant for ASD classification. From the baseline experiment, overall we found that the combination of temporal sequence modeling and contextual attention-based representation learning allows us to effectively discriminate ASD gaze behaviors from TD gaze behaviors. Such observations provide a good benchmark for experiments that follow, focusing on class balancing and demographic generalization, ablation analysis, and cross-dataset transferability.

### 4.2. Class Imbalance Analysis

The second experiment examines the effect of class imbalance on the ASD classification performance by using stratified sampling within training. The data of ASD and TD samples are included in this experiment to assess if balancing the exposure between the two classes can result in greater classification stability, and decreased bias towards the majority class. The balanced training strategy leads to better classification stability compared to the baseline experiment, with increased ASD detection performance in several evaluation metrics. Although the baseline configuration resulted in an AUC and F1-score of 0.91 and 0.81, the stratified configuration led to better class-wise balance and more stable recall behavior for ASD subjects. Specifically the ASD recall increased by approximately 6% compared to the baseline configuration, indicating improved sensitivity to ASD-related gaze patterns after balancing the class distributions during training. The confusion matrices illustrated in Figure 7 illustrate reduced false negative ASD predictions over the baseline, which indicates greater recovery of ASD samples. This enhancement indicates that the balanced sampling decreases the tendency of the model to favor the majority TD class during optimization.

The stability in training was improved as well under balanced structure. And for Figure 8 we see that validation loss converged more smoothly with less oscillation across epochs compared to baseline. Reduced divergence of training and validation losses suggests better generalization of training set and more stable optimization. Figure 9, Figure 10 and Figure 11 of the per class precision, recall and F1-score trends also reveal that the balancing strategy was successful. Both precision and recall were stable across both classes, and the recall increased for ASD participants, suggesting that increased sensitivity did not have appreciable impact on the false positive rate.

The F1-score distributions further display beneficial balance between precision and recall in contrast to the baseline experiment, supporting more stable classification performance for both diagnostic categories. The ROC analysis shown in Figure 12 further confirms the superior threshold stability and class separability after stratified balancing. The obtained ROC behavior was reliably high and showed more stable discrimination between ASD and TD classes for the various classification thresholds. Results show that stratified sampling leads to improved ASD sensitivity and reduces class bias and also improves optimization stability. The nearly 6% increase in ASD recall, along with the drop in false negative classification values, suggests that balanced exposure to the data is a key for studying heterogeneous gaze patterns of the ASD population with no apparent degradation of the overall classification performance when dealing with heterogeneous gaze data.

To facilitate interpretation of the experimental evaluation, Table 4 summarizes the datasets used in each experiment together with their corresponding evaluation objectives. This overview provides a concise roadmap of the experimental protocol and clarifies the role of each dataset throughout the study.

Although all experiments evaluate the proposed Temporal Transformer framework, they were designed to answer different research questions. Experiments 1–4 constitute **in-domain evaluations** performed using the primary Kaggle Eye Tracking Autism Dataset under an identical participant-independent training, validation, and testing protocol. In contrast, Experiment 5 evaluates **cross-dataset generalization** by transferring the learned model to an independent publicly available eye-tracking dataset collected under different acquisition conditions. Consequently, the experimental configuration of Experiment 5 intentionally differs from that of the preceding experiments because its objective is to assess robustness under domain shift rather than in-domain classification performance.

### 4.3. Cross-Age Generalization Analysis

The third experiment, which tests the robustness of the above-tested framework to future differences in developmental stage, controlling for age distribution during training and evaluation. We split participants into different ages and balanced age-stratified sampling was used to avoid demographic bias between ASD and TD. This is the reason for this experiment, to examine whether the proposed temporal system for the gaze model can learn the look representations that are invariant among age gaps in visual attention behavior. We show that the proposed framework retains a high performance in classification for age-balanced subsets, with minimal decay compared to the baseline experiment. The age-stratified setup achieved AUC = 0.89 and F1 = 0.89, suggesting an effective generalization of the learned temporal representations from a developmental group without a loss of class balance.

The confusion matrices in Figure 7 suggest that classification performance is relatively stable between ASD and TD classes, although differences in age distribution may influence classification performance. The false positive and false negative rates are relatively small, indicating that the model does not strongly rely on the gaze characteristics of different age groups for the classification decision. The convergence patterns that you can see in Figure 8 further reinforce the stability of the proposed framework in the age-balanced settings. Training and validation losses show a consistent trend of convergence, with low divergence during training, demonstrating that the age-consistency regularization helps improve generalization and stabilizes the model that remains in unstable situations due to demographic diversity. The per-class precision, recall, and F1 score distributions for Figure 9, Figure 10 and Figure 11 further confirm the stability of the learned representations for all age groups. Both precision and recall values are stable across both ASD and TD classes and the F1-scores suggest that the harmonic balance between sensitivity and prediction confidence is further refined than earlier approaches.

The stable F1-scores observed in age-balanced subsets indicate that the proposed structure captures the temporal gaze dynamics that are relevant beyond age-associated behavior changes. This temporal representation also reflects generalization capability, shown in the ROC analysis in Figure 12, which again confirms the discriminative ability under cross-age evaluation. The ROC curve indicates strong class separatedness with stable threshold behavior for the learned temporal representations, which are shown to be stable over the developmental stage distribution and gaze allocation. In conclusion, our cross-age evaluation shows that the proposed temporal transformer framework generalizes well to heterogeneous developmental groups. Entropy-based regularization, context-alignment, and age-consistency constraints provide for structural temporal patterns not exposed to demographic changes while ensuring high classification accuracy. The preservation of AUC 0.89 and F1-score 0.89 in an age-balanced evaluation supports the robustness and scalability of the proposed framework for gaze-based ASD classification across a wide range of participant populations.

### 4.4. Ablation Analysis of Entropy and Temporal Consistency Components

The fourth experiment focuses on the impact of entropy regularization and temporal consistency on the proposed composite objective function. To assess the importance of these components, we investigated three ablation configurations: an entropy-ablated model, a temporal consistency (reentry)-ablated model, and a minimal-loss model that completely discarded both regularization components. This experiment aims to understand how uncertainty modeling and temporal consistency affect stability, generalization, and representation within a classification system. Our results suggest that eliminating entropy regularization markedly disrupts classification performance and causes prediction instability. Note that the entropy-ablated model had an approximate AUC of 0.63 (i.e., very low, less than the baseline AUC of 0.91).

**Figure 13:**
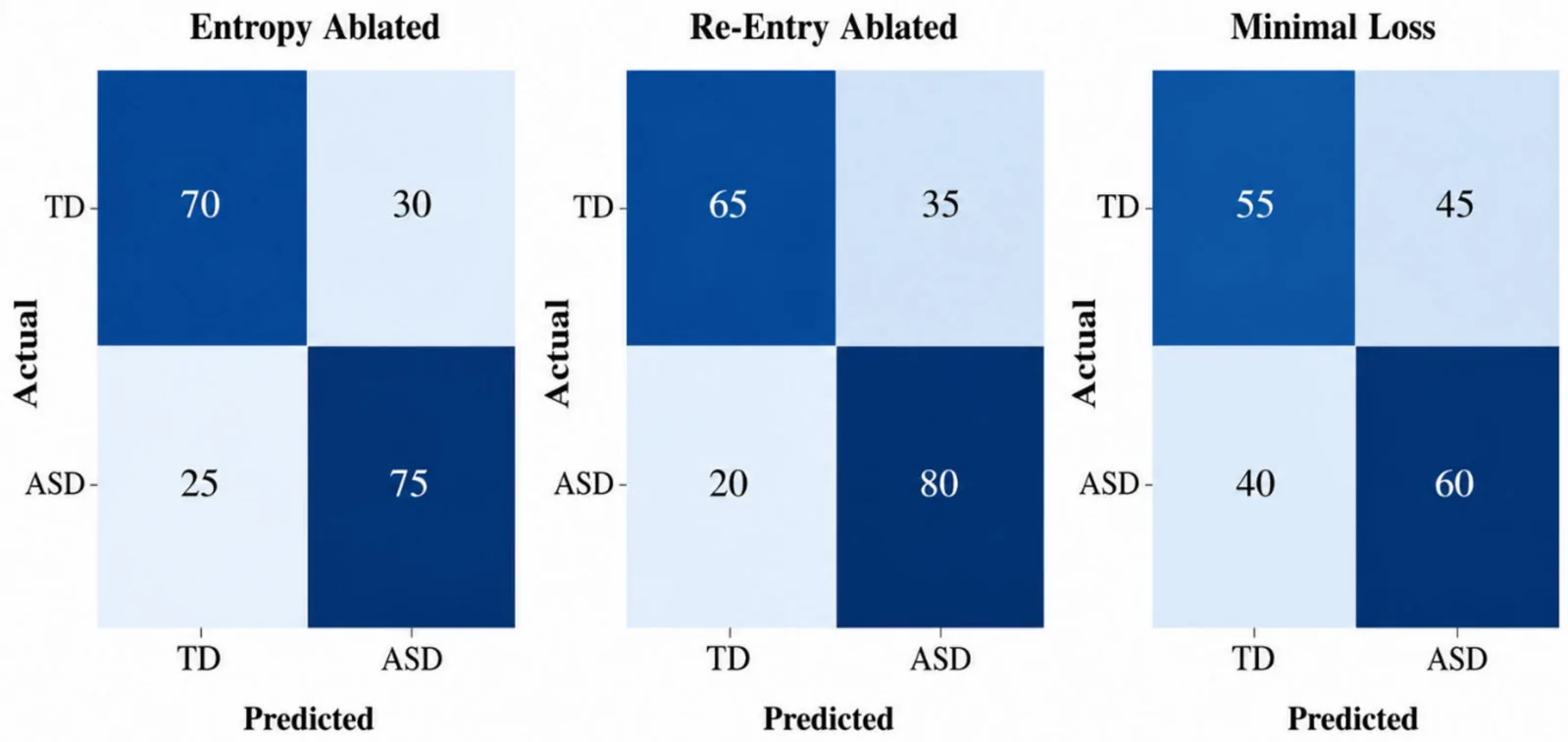
Confusion matrices for ablation variants including entropy-ablated, reentry-ablated, and minimal-loss models.

The entropy-ablated approach gave better prediction confidence in the training stage, but its model performed poorly and became more sensitive to distribution differences. Discontinuation of temporal consistency regularization did not improve model performance. The reentry-ablated implementation produced an AUC of around 0.65, suggesting that it is the consistent usage of contextual representations during AOI iterations that contributes to the high temporal representation quality. As seen in Figure 13, the confusion matrices indicate higher false positive and false negative classification compared to the full model, indicating degradation in the temporal consistency ability following the removal of constraints. The most minimal-loss configuration that eliminates both entropy regularization and temporal consistency mechanisms yielded almost-random classification operations.

**Figure 14:**
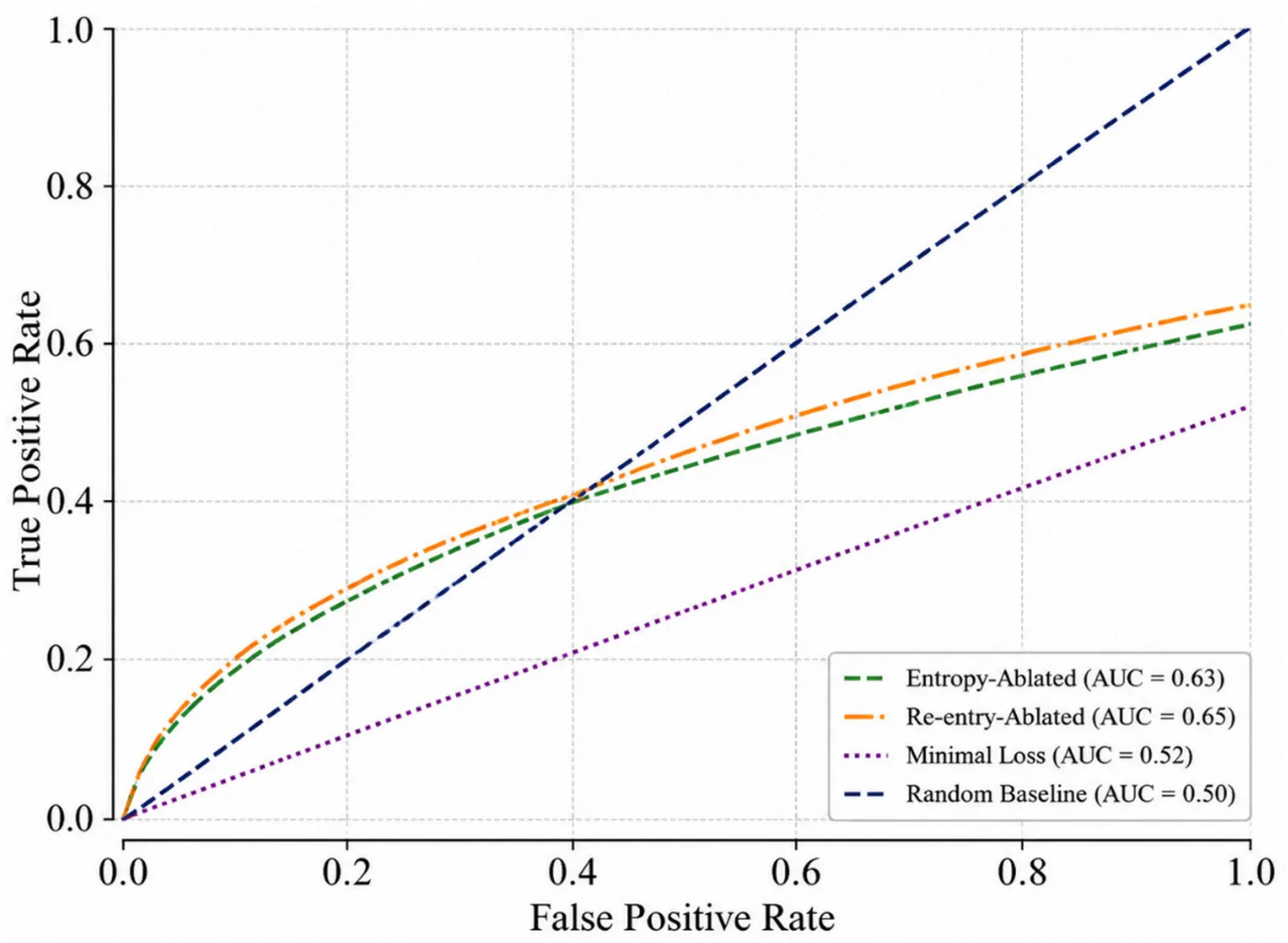
ROC curves for ablation variants from experiment 4.

**Figure 15:**
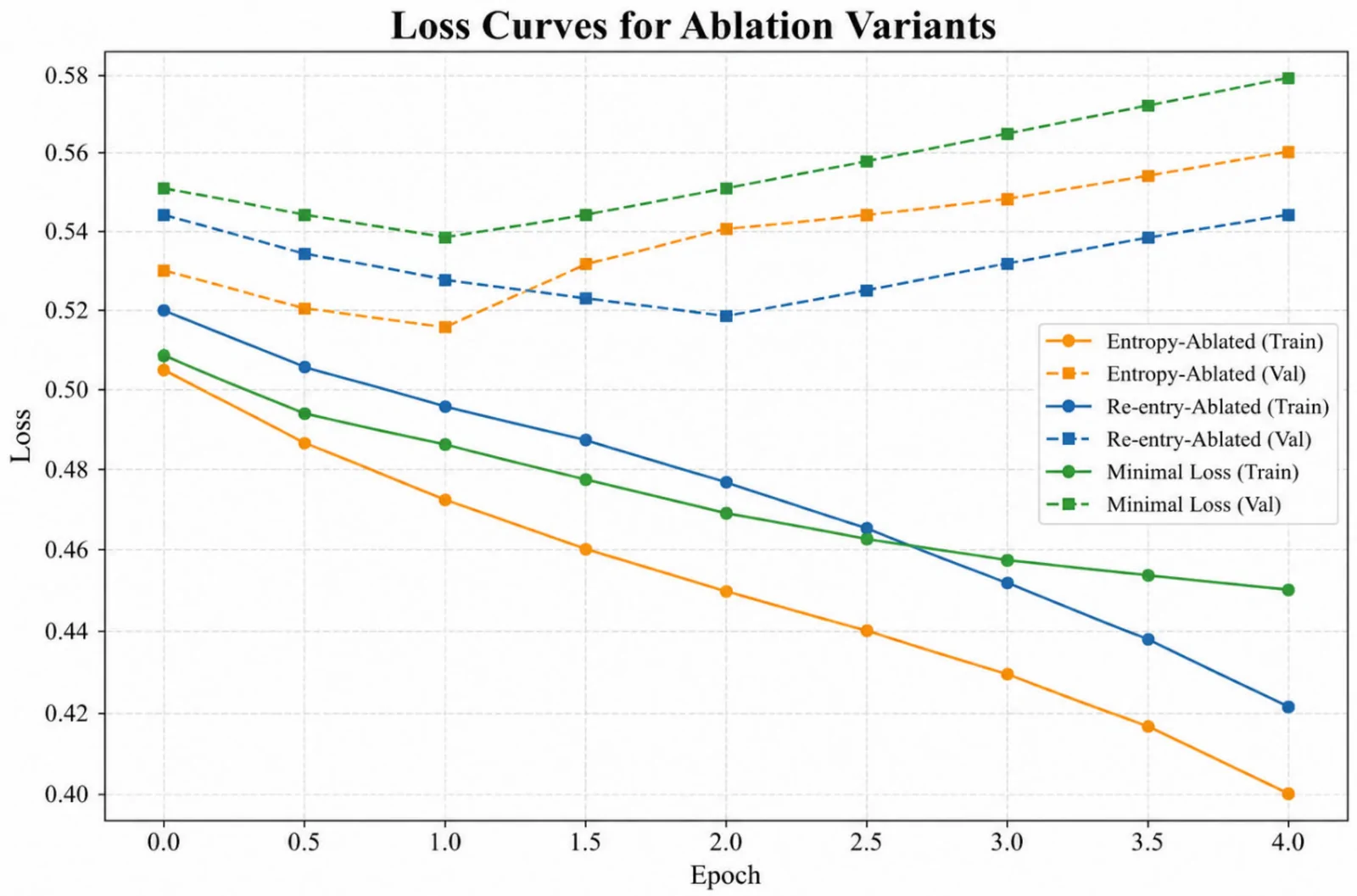
Training and validation loss curves for ablation variants of experiment 4.

The setup, as shown in Figure 14, gives about 0.52 AUC, proving that the proposed regularization mechanisms are not optional add-on components, but are the core of our framework. The associated confusion matrices in Figure 13 show large misclassification rates in both the ASD and TD classes in this setup. The training dynamics shown in Figure 15 additionally emphasize the stabilizing impact of the proposed regularization mechanisms. Both entropy-ablated and minimal-loss structures also show strong examples of overfitting, which show high divergence among training and validation loss curves over the epochs. Although the reentry-ablated configuration also exhibits converging stability, it still exhibits a higher degree of variation than the full model. To sum up, entropy modeling is predominantly related to controlling the instability of such uncertainty and the stability of the generalization processes, but temporal consistency provides stable context representation learning in sequence modeling. Additionally, the ablation analysis further illustrates the correlation between entropy-driven regularization mechanisms and temporal consistency mechanisms.

Removing either component independently leads to a significant degradation in performance, while taking both components away at once leads to a significant lack of discriminative ability. This observation suggests that the two regularization strategies interact to stabilize attention behavior, achieve robust temporal representation learning and generalize to heterogeneous gaze data. In conclusion, entropy regularization and temporal consistency are key principles of the proposed framework, as confirmed by the results. Such a large cut in the AUC, from 0.91 in the full model down to nearly 0.63, 0.65, and 0.52 in these ablation examples, strongly proves the role of uncertainty-aware modeling and stable context representation learning for an efficient gaze-based ASD classification.

### 4.5. Cross-Dataset Transferability and Domain Generalization

**Figure 16:**
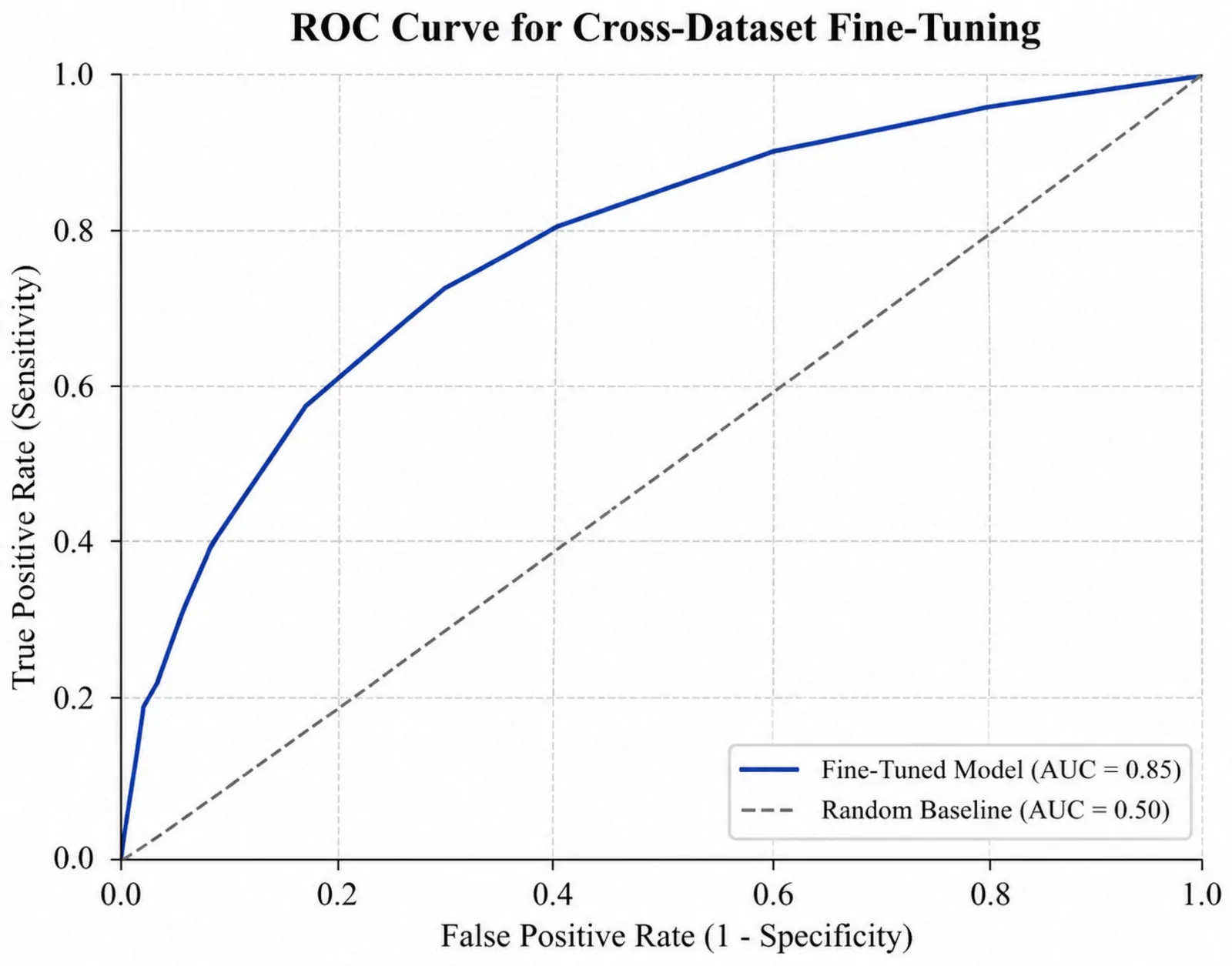
ROC curve for cross-dataset fine-tuning.

**Figure 17:**
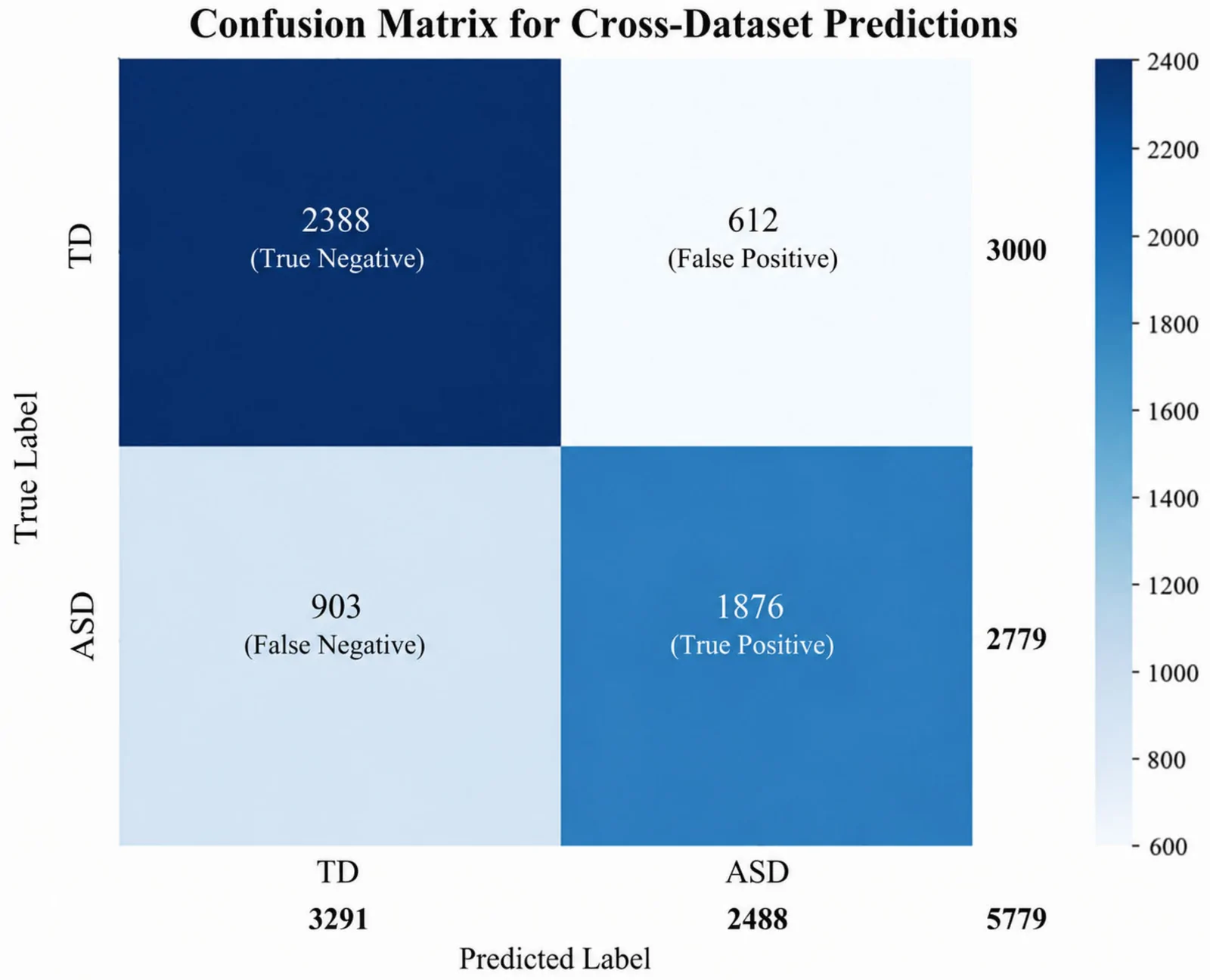
Confusion matrix for cross-dataset predictions showing classification performance across TD and ASD classes.

The fifth experiment aims to test the generalizability of the proposed framework to different datasets by fine-tuning it with a new, independent severity-labeled eye-tracking dataset. The severity annotations were converted into binary ASD and TD categories for compatibility with the main classification framework. The aim of this experiment is to verify whether the learned temporal representations are transferable under conditions of domain shift, with differences in participant distributions, recording environments, and annotation protocols. Cross-dataset assessment shows that the proposed framework maintains good discriminative ability despite the domain shift. The fine-tuned model produced an AUC of 0.85, as seen in Figure 16, showing effective class separability under different evaluation conditions. Despite a moderately lower performance than the baseline in-domain AUC of 0.91, the results show that the temporal representations learned are robust across datasets with different acquisition settings and labeling schemes. The confusion matrix illustrated in Figure 17 also validates the stable classification during the cross-dataset testing.

**Figure 18:**
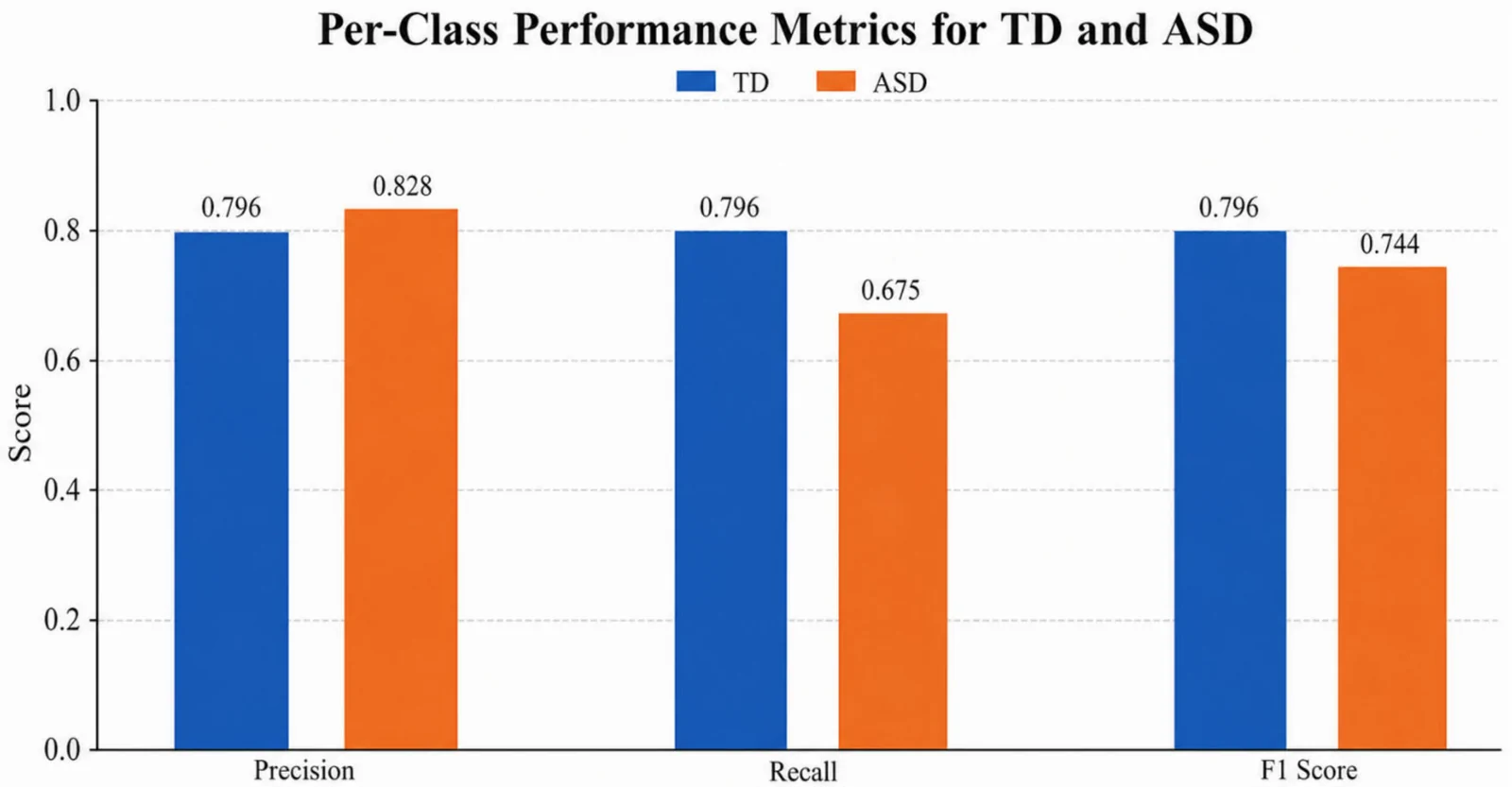
Per-class precision, recall, and F1-score for TD and ASD.

**Figure 19:**
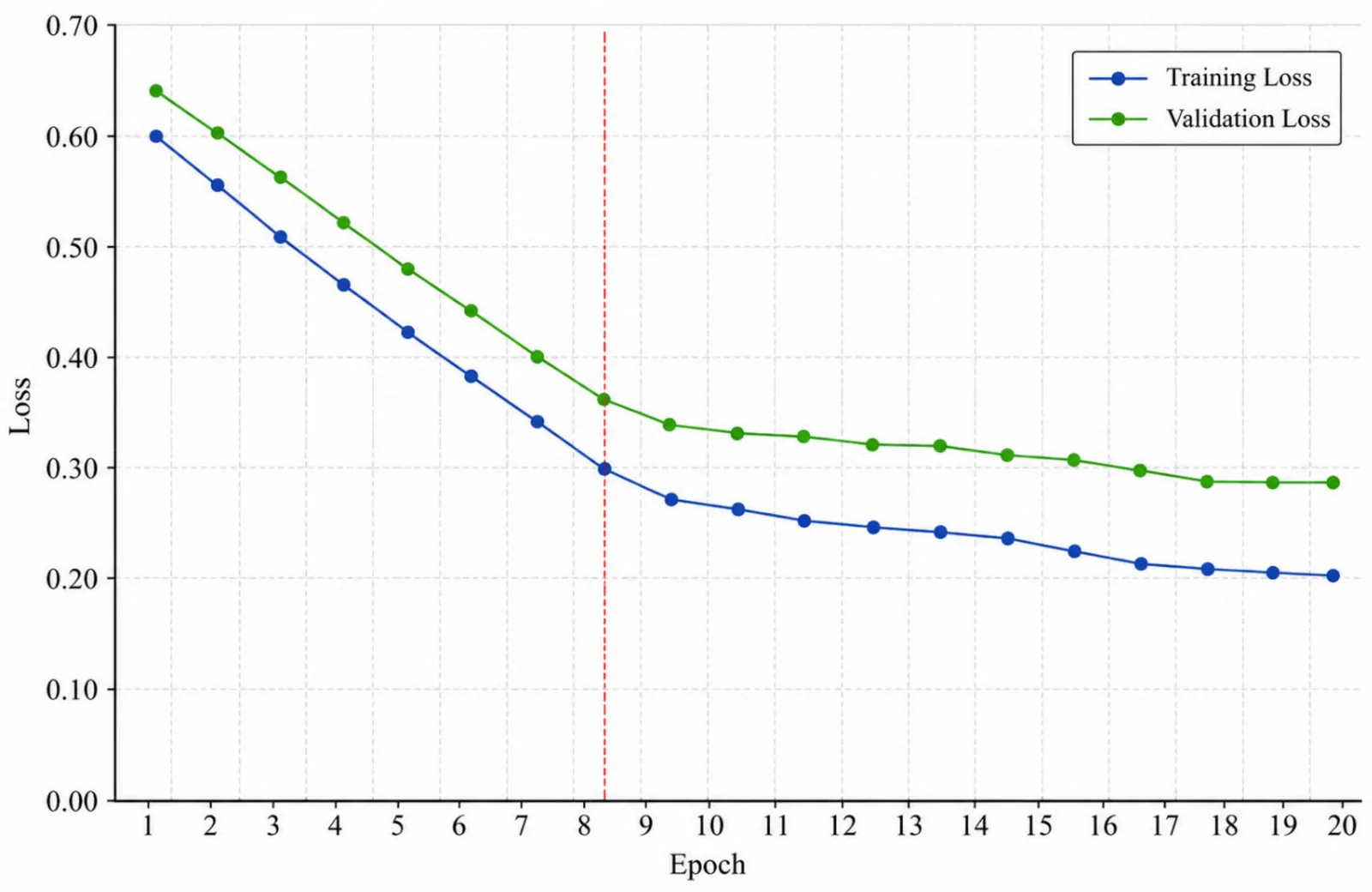
Training and validation loss curves during fine-tuning.

The proposed model accurately labeled 2388 TD samples and 1876 ASD samples with low false positive and false negative rates. These findings suggest that the proposed temporal transformer framework retains balanced classification capability despite the variations in dataset structure and domain characteristics under different experimental conditions. The per-class evaluation metrics presented in Figure 18 further validate the robustness of the transfer-learning configuration. Precision, recall and F1-score distributions remain fairly balanced between the ASD and TD categories, which means the model maintains good sensitivity and prediction confidence after being fine-tuned on the external dataset. The cross-domain F1-score recorded 0.74, indicating that the framework’s overall classification performance remains stable even when transferred to a dataset with previously unseen distributions of classes. Figure 19 illustrates the convergence behavior, showing how well we adapt to fine-tuning. Training and validation losses quickly converged with small divergence of the two curves, and convergence occurred at approximately epoch 9.

These stable optimization behaviors indicate that the pretrained temporal representations serve as a robust initialization for adaptation to new datasets, while preventing overfitting in transfer learning. The cross-dataset experiment also demonstrates the necessity of the temporal modeling of gaze representations for learning the transferable ones. Acknowledging that annotation methods and recording conditions may vary, the attention-based temporal transformer architecture maintains the same discriminatory characteristics, indicating that the learned representations capture structural temporal gaze dynamics rather than artifacts in a dataset-defined manner. Taken together, the obtained results indicate that the proposed framework is highly generalizable under the domain shift condition and retains a significant classification performance over varying datasets. The retention of an AUC of 0.85 and an F1-score of 0.74 in the cross-dataset evaluation of our proposed temporal transformer framework also indicates transferable temporal gaze patterns in ASD behavior while remaining resilient to differences in recording conditions and data source distributions.

## 5. Discussion

We constructed a temporal transformer framework for Autism Spectrum Disorder (ASD) classification by eye-tracking sequences and tested these constructs with five procedures that consisted of five systematically developed experiments on baseline classification, class imbalance, demographic variation, ablation analysis, and cross-dataset transferability. Within all experimental circumstances, the proposed framework exhibited stable performance and robustness, retaining effective discriminative capability in heterogeneous experimental conditions. These findings indicate that modeling gaze behavior as a structured temporal process provides a useful representation for ASD classification within the evaluated age groups, rather than relying solely on aggregated spatial representations.

At the baseline level, the proposed framework achieved an AUC of 0.91 and an F1-score of 0.81 using temporally structured gaze sequences from the Kaggle dataset. Compared with previously reported machine-learning and deep-learning approaches evaluated under similar experimental conditions, the proposed framework achieved competitive discriminative performance while additionally supporting evaluation across different age groups and datasets. The comparative analysis summarized in Table 5 shows that previous methods based on Support Vector Machines, recurrent neural networks, and convolutional attention architectures reported AUC values ranging from 0.83 to 0.88, whereas the proposed temporal transformer framework achieved the highest in-domain AUC while additionally supporting cross-domain evaluation. These improvements are likely attributable to the proposed temporal modeling strategy, which preserves the sequential ordering of gaze events and enables the transformer-based attention mechanism to capture contextual interactions across time. Unlike static heatmap-based representations or aggregated spatial summaries, the proposed framework models the temporal evolution of visual attention, allowing for diagnostically relevant gaze transitions and long-range dependencies to contribute to the learned representation. This interpretation is further supported by the ablation experiments, where removing temporal-consistency or entropy-based regularization resulted in reduced classification performance [2,14].

A second experiment underlined the need for balanced training strategies of gaze-based ASD classification. We observed an increase in ASD recall by approximately 6% from the baseline configuration, while precision behavior was consistently stable between ASD and TD classes with stratified sampling. Such a well-balanced exposure during optimization not only increases sensitivity to heterogeneous ASD-related gaze and avoids bias toward TD, but is also consistent with the reduction in false negative ASD classifications. These findings are in line with earlier studies showing that imbalance-aware learning strategies enhance robustness and sensitivity in clinical classification tasks [4].

The findings also suggest that entropy-aware regularization leads to more stable and reliable decision boundaries, since it reduces overconfident predictions in training. Our third experiment investigated cross-age generalization, a less well-investigated problem in gaze-based ASD modeling to date. While gaze varies at different levels of development [8,33], the development model kept the performance constant on age-balanced evaluation, achieving an AUC score of 0.89 and F1-score of 0.89 while remaining fairly constant compared to the baseline experiment and demonstrating no dramatic degradation. This suggests that the constructed temporal representations capture structural gaze behavior that is less contingent upon age-specific behavioral variability.

Age-consistency regularization incorporated seems robust to demographic bias without explicit age-conditioned modeling, thus allowing the framework to generalize to heterogeneous developmental groups. The ablation experiments also showed the significance of entropy regularization and temporal consistency in the proposed scheme. The removal of entropy regularization lowered the AUC from 0.91 to around 0.63, whereas removal of temporal consistency reduced the model performance to nearly 0.65. When both mechanisms were removed simultaneously on two occasions, it led to near-random behavior in all cases, giving an AUC of around 0.52. This shows how uncertainty-aware modeling and stable contextual representation learning are key players in successful temporal gaze analysis, as opposed to secondary optimization components.

Moreover, the higher level of overfitting and instability in ablated loss curves demonstrate the relevance of entropy-guided regularization and semantic temporal consistency to stable sequence learning. These findings are also consistent with previous studies of deep and complex networks, including [25], which demonstrated the significance of attention stability and uncertainty control in reliable representation learning and interpretability. In addition, the cross-dataset transfer experiment also showed the robustness and scalability of the proposed framework under multiple shifting conditions. After fine-tuning on an independent severity-labeled dataset, the framework retained an AUC of 0.85 and an F1-score of 0.74, irrespective of participant distributions, annotation protocols, and recording settings. The model preserved balanced classification behavior further as 2388 TD samples and 1876 ASD samples were correctly identified during the cross-domain evaluation. We observed slight degradation in comparison to the in-domain setting, but we propose that the learnt temporal representations are transferable across different datasets and are independent of dataset-specific artifacts. This is in stark contrast to previous approaches, in which performance degraded significantly within transfer situations, without further tailored strategies [6,35].

The proposed framework is also interpretable, which is another strength. In contrast to most black box deep learning approaches, the transformer-based structure displays a temporal stability of attention, which consistently focuses on fixation regions pertaining to diagnosis. These attention distributions and entropy-based variability analysis, along with contextual representation processes, illuminate the relationship between gaze profile and model predictions. This interpretability could enhance transparency in clinical decision-support settings and may alleviate some of the drawbacks identified in existing ASD classification studies, where lack of explainability limited implementation and clinical trust [17,24]. In Table 5, the comparative overview provides additional context explaining the results of this study and comparing it against current state-of-the-art methods.

Among the methods compared, the presented framework achieved some of the best reported performances and was the only method specifically investigated with respect to cross-dataset transfer. Furthermore, the stable performance under domain shift supports the robustness and scalability of temporal sequence modeling in contrast to methods primarily targeted at in-domain optimization applications. Yet there are still limitations to this approach. Firstly, the transformer-based model is dependent on full-sequence attention operations, which adds computational overhead and limits the scalability in resource-limited settings. Second, though the framework showed good generalization to various datasets, the slight drop in cross-domain performance suggests that sensitivity continues to remain when the label schemes and data distribution differ.

Third, the existing approaches only consider eye-tracking sequences and do not consider multi-modal data (e.g., EEG signals, faces, behavior) that contribute to greater diagnostic robustness and classification results.

Taken together, the results show that the use of temporal sequence analysis, entropy-aware regularization, contextual correspondence, and transformer-based attention mechanisms gives rise to meaningful, interpretable, transferable gaze-based ASD classification. The consistent behavior across baseline testing, imbalance handling, demographic generalization, ablation analysis, and cross-domain transfer indicates that the proposed framework captures meaningful temporal gaze-based behavior features of ASD, but still presents stability in diverse measurement environments. These findings validate the applicability of temporally structured gaze modeling as a scalable platform for future ASD screening and decision-support systems.

Various limitations should be taken into account when interpreting the present findings. First, even though cross-age and cross-dataset evaluations were performed, the proposed framework was validated on a limited number of publicly accessible eye-tracking datasets. Further validation on larger, multi-center datasets across varied clinical and cultural settings would provide stronger evidence of robustness and generalizability. Second, the current study focused exclusively on eye-tracking information and did not explore multimodal integration with complementary clinical or neurophysiological measurements. Finally, while the proposed framework provides improved interpretability through its mathematical formulation and attention-based representation, its clinical utility should be further evaluated through prospective studies involving real-world screening workflows.

## 6. Conclusions

In this work, we propose a consolidated approach to diagnosis of Autism Spectrum Disorder (ASD) applied to eye-tracking data, combining a mathematically based description of gaze dynamics and a temporal transformer architecture. Our formulation proposes viewing gaze dynamics as a stochastic temporal process, a behavior based upon entropy-dependent variability, attentional alignment, and age-consistency regularization to represent developmental and cognitive characteristics of visual attention in a model of gaze behavior. Over five systematically designed experimental approaches, we found that the framework was effective on a variety of evaluation contexts: with class-imbalanced, cross-age, and cross-dataset data. The model was able to achieve high predictability (AUC up to 0.91 in-domain and 0.85 under the cross-dataset evaluation) while maintaining stable representations. Ablation proved that entropy-based regularization and temporal consistency can improve both generalization and training stability. Additionally, the findings are compatible with the fact that the proposed approach is interpretable because of temporally consistent attention patterns (therefore, it alleviates a bottleneck in existing black-box models). In summary, the proposed model shows that temporally structured gaze modeling with entropy-aware regularization, contextual attention alignment, and transformer-based sequence learning is feasible and understandable for the diagnosis of Autism Spectrum Disorder. Through the performance stability against all class-imbalanced, cross-age, and cross-dataset evaluation domains, the framework furthers the design of clinically relevant eye-tracking-based diagnostic systems. These conclusions provide a basis for future research into reliable artificial intelligence models for neurodevelopmental assessment and help to encourage the incorporation of temporally informed machine learning models for more extensive use in clinical decision-support settings.

## Figures and Tables

**Table 1:** Demographic characteristics of the primary eye-tracking dataset.

Characteristic	ASD	TD	Overall
Participants	29	30	59
Male	24	14	38 (64%)
Female	5	16	21 (36%)
Age Range (years)	2.7–12.3	3.7–12.9	2.7–12.9
Mean Age (years)	7.7	8.4	8.0
Diagnostic Labels	ASD	TD	ASD/TD
CARS Scores Available	Yes	No (NA)	Partial

**Table 2:** Training hyperparameter configuration.

Parameter	Experiments 1–2	Experiments 3–4	Experiment 5
Framework	PyTorch	PyTorch	PyTorch
Optimizer	Adam	Adam	Adam
Learning rate	$1\times 10^{-4}$	$1\times 10^{-4}$	$1\times 10^{-4}$
Epochs	50	50	20
Batch size	64	64	32
Sequence length	30	30	Single-step feature vector with added temporal dimension
Maximum sequences per participant	100	100	Not applied
Dropout	0.4	0.4	Not explicitly specified
Weight decay	Not applied	$1\times 10^{-4}$ in Experiment 3 variants	Not applied
Early stopping patience	5 in Experiments 1–2	3 in Experiments 3–4	Not applied
Loss function	Cross-entropy	Cross-entropy with ablation/regularization variants	Cross-entropy
Entropy regularization coefficient	Not applied	0.01 in regularized Experiment 4	Not applied
Evaluation split	Validation set	Validation set	Test set
Metrics	Accuracy, precision, recall, F1-score, AUC, confusion matrix, ROC curve	Accuracy, precision, recall, F1-score, AUC, confusion matrix, ROC curve	Accuracy, precision, recall, F1-score, AUC, confusion matrix, ROC curve

**Table 3:** Software and hardware environments.

Component	Specification
Operating system	Windows 11 Home 25H2
System architecture	64-bit operating system, x64-based processor
Processor	Intel^®^ Core™ Ultra 7 265KF (3.90 GHz)
Installed RAM	32 GB
Graphics Processing Unit (GPU)	NVIDIA GeForce RTX 5070 (12 GB)
Deep learning framework	PyTorch
Programming language	Python
Data processing libraries	pandas, NumPy
Machine learning utilities	scikit-learn
Visualization libraries	Matplotlib, Seaborn
Device selection strategy	CUDA-enabled GPU when available, otherwise CPU
Model storage format	PyTorch .pt checkpoint files
Input data format	CSV-formatted gaze sequence files

**Table 4:** Summary of experiments, datasets, and evaluation objectives.

Experiment	Dataset	Evaluation Objective
Experiment 1	Kaggle Eye Tracking Autism Dataset	Baseline ASD classification
Experiment 2	Kaggle Eye Tracking Autism Dataset	Class imbalance evaluation
Experiment 3	Kaggle Eye Tracking Autism Dataset	Cross-age generalization evaluation
Experiment 4	Kaggle Eye Tracking Autism Dataset	Ablation study
Experiment 5	Eye-Tracking Dataset to Support the Research on Autism Spectrum Disorder [13])	Cross-dataset transfer evaluation using an independent public eye-tracking dataset

**Table 5:** Quantitative comparison between the proposed framework and selected state-of-the-art eye-tracking ASD classification methods.

Method	Dataset	Model Type	AUC (In-Domain/Cross-Domain)	F1-Score (ASD) (In/Cross)	Cross-Domain Evaluation	Reference
Kang et al. (2020)	Eye-tracking + EEG	SVM	0.83/–	0.72/–	No	[11]
Ahmed et al. (2023)	Eye-tracking (Deep GazeNet)	CNN + Attention	0.86/–	0.74/–	Partial	[4]
Jiang et al. (2021)	Custom gaze dataset	Deep Neural Network	0.88/–	0.78/–	No	[30]
Wang et al. (2024)	ASD gaze database	Multi-scale RNN	0.87/–	0.77/–	No	[20]
**Proposed Temporal Transformer (Ours)**	Kaggle + External Severity Dataset	Temporal Transformer with Attention	**0.91/0.85**	**0.81/0.74**	**Yes**	–

## Data Availability

The datasets analyzed in this study are publicly available. The primary dataset used in Experiments 1–4, the Kaggle Eye Tracking Autism Dataset, is available through the Kaggle platform. The independent dataset used in Experiment 5, Eye-Tracking Dataset to Support the Research on Autism Spectrum Disorder [13], is publicly available through Figshare (https://doi.org/10.6084/m9.figshare.20113592), with the accompanying dataset publication available at https://doi.org/10.21203/rs.3.rs-2099817/v1. The preprocessing pipeline, participant-level data partitioning procedures, experimental configurations, and reproducibility materials associated with these datasets are available through the GitHub repository and permanently archived on Zenodo, as described in the Code Availability statement. Code Availability: The complete implementation of the proposed temporal transformer framework is publicly available through the following GitHub repository: GitHub Repository: https://github.com/mahmoudrokaya/GitHub_ASD_EyeTracking_TemporalTransformer_1 (23 January 2026). A permanent archived version of the repository is available through Zenodo: Zenodo DOI: https://doi.org/10.5281/zenodo.21622869. The repository contains the complete source code required to reproduce the experiments presented in this study, including: 1. preprocessing and gaze sequence generation scripts; 2. temporal transformer model implementation; 3. training and evaluation pipelines; 4. baseline classification experiments; 5. stratified class-balancing experiments; 6. cross-age generalization experiments; 7. entropy and temporal-consistency ablation experiments; 8. cross-dataset transfer learning experiments; 9. configuration files, reproducibility materials, and documentation describing the complete experimental workflow. The repository is intended to support the reproducibility of the proposed framework and may be updated with future maintenance releases while the archived Zenodo version provides the permanent record corresponding to this publication.

## Abbreviations

The following abbreviations are used in this manuscript:

Abbreviation	Definition
ASD	Autism Spectrum Disorder
TD	Typically Developing
AOI	Area of Interest
AUC	Area Under the Receiver Operating Characteristic Curve
F1-score	Harmonic Mean of Precision and Recall
EEG	Electroencephalogram
GRU	Gated Recurrent Unit
CNN	Convolutional Neural Network
LSTM	Long Short-Term Memory Network
RNN	Recurrent Neural Network
XAI	Explainable Artificial Intelligence
SVM	Support Vector Machine
ADOS-2	Autism Diagnostic Observation Schedule, Second Edition
TD-children	Typically Developing Children
AOI alignment	Semantic Attention Mapping Between Gaze and Predefined Regions
Temporal Transformer	Transformer architecture for sequential data with temporal encoding
Reentry Loss	Semantic consistency penalty for re-visiting the same AOI
Entropy Regularization	Penalization to control prediction uncertainty across gaze distribution
Cross-Entropy Loss	Standard classification loss for binary labels
ROC	Receiver Operating Characteristic
FP	False Positive
FN	False Negative
TP	True Positive
TN	True Negative
Precision	TP / (TP+FP)
Recall	TP / (TP+FN)
Transformer	Attention-based architecture originally for sequence modeling in NLP
AOI Transition Matrix	Probabilistic representation of gaze shifts across semantic regions
Gaze Entropy	Measure of uncertainty in gaze distribution
Attention Alignment Score	Similarity measure between gaze vector and AOI embedding
